# *AlignPCA*-2*D*: PCA-reduced Euclidean vector alignment for 2D classification in cryo-EM

**DOI:** 10.1107/S2059798326004572

**Published:** 2026-06-08

**Authors:** E. Ramírez-Aportela, O. L. Zarrabeitia, Y. C. Fonseca, T. Ceska, S. Subramaniam, J. M. Carazo, C. O. S. Sorzano

**Affiliations:** a Centro Nacional de Biotecnología (CSIC), C/Darwin 3, 28049Cantoblanco, Madrid, Spain; b Gandeeva Therapeutics Inc., Vancouver, British Columbia, Canada; c Department of Biochemistry and Molecular Biology, University of British Columbia, Vancouver, British Columbia, Canada; National Centre for Biological Sciences-TIFR, India

**Keywords:** 3D reconstruction and image processing, single-particle cryoEM, *AlignPCA*-2*D*

## Abstract

Cryogenic electron microscopy (cryo-EM) has transformed structural biology by enabling the high-resolution reconstruction of macromolecular complexes from noisy projection images. However, the intrinsic heterogeneity and low signal-to-noise ratio of cryo-EM datasets make 2D classification a critical and computationally demanding step in the processing workflow. Here, we introduce *AlignPCA*-2*D*, a principal component analysis (PCA)-space Euclidean vector alignment method for fast, interpretable 2D classification in cryo-EM. By projecting particle images and class representations into a compressed latent PCA space, *AlignPCA*-2*D* reduces data dimensionality while preserving meaningful structural variability. The image-to-class assignment is then performed using Euclidean distance, enabling efficient and accurate classification. We benchmark *AlignPCA*-2*D* against established cryo-EM software, such as *RELION* and *CryoSPARC*, and demonstrate that it achieves competitive alignment accuracy while substantially reducing computational cost. This approach provides a lightweight alternative for large-scale 2D classification tasks, and its modular design makes it compatible with existing cryo-EM processing pipelines.

## Abstract 2

We introduce *AlignPCA*-2*D*, a PCA-space Euclidean alignment method for rapid and interpretable 2D classification of cryo-EM particle images. The approach preserves essential structural variability while greatly reducing computational cost, offering a lightweight alternative to existing large-scale cryo-EM classification pipelines.

## 1. Introduction

Cryogenic electron microscopy (cryo-EM) has emerged as a transformative technique for resolving the structures of biological macromolecules under near-native conditions. By capturing two-dimensional projections of molecules frozen in vitreous ice, cryo-EM provides insights into structural dynamics that are often inaccessible by other methods. However, raw cryo-EM datasets typically contain heterogeneous mixtures of particle conformations and orientations, along with significant levels of noise, making the extraction of meaningful structural information a considerable computational challenge. Two-dimensional (2D) classification plays a pivotal role in cryo-EM image-processing pipelines. It groups similar particle images based on shared structural features, enhancing the signal-to-noise ratio and revealing distinct molecular states and orientations. This step improves data interpretability and serves as a crucial foundation for subsequent processes, such as generating *ab initio* 3D models and refining the volume.

A central difficulty in 2D classification arises from the high dimensionality of cryo-EM images, which can contain hundreds of thousands of pixels per particle. Analyzing these data directly is computationally intensive and susceptible to overfitting. To mitigate these challenges, dimensionality-reduction techniques, particularly principal component analysis (PCA), have been widely adopted.

The application of these methods has a long-standing history, beginning with the multivariate statistical analysis (MSA) framework introduced by van Heel & Frank (1981 ▸). These pioneering approaches, later implemented in software such as *SPIDER* (Frank *et al.*, 1996 ▸) and *IMAGIC* (van Heel *et al.*, 1996 ▸), utilized eigenvector-based decomposition in the real-space domain to extract structural variability from noisy projections. While modern maximum-likelihood and Bayesian formulations, such as those implemented in *RELION* (Scheres, 2012 ▸) and *CryoSPARC* (Punjani *et al.*, 2017 ▸), currently dominate high-resolution refinement, the core principles of MSA (linear dimensionality reduction and variance-driven separation of structural states) remain computationally attractive, especially for handling the massive datasets generated by modern detectors. In this context, PCA provides a natural way to project high-dimensional image data onto a lower-dimensional subspace that captures the dominant modes of variability while suppressing noise and redundancy.

To quantify structural similarity among images, different metrics have been explored, ranging from classical cross-correlation and Euclidean distance to more complex formulations such as Mahalanobis covariance-based metrics (Bhamre *et al.*, 2017 ▸) and Wasserstein optimal transport distances (Rao *et al.*, 2020 ▸). While cross-correlation has traditionally been employed in cryo-EM alignment, Euclidean distance in a reduced latent space provides a computationally efficient alternative for large-scale datasets. Notably, when the compared images are normalized to have equal norm, maximizing cross-correlation is equivalent to minimizing the Euclidean distance.

In this work, we introduce *AlignPCA*-2*D*, a novel method for 2D classification in cryo-EM that combines PCA-based compression of the Fourier representation of particle images with Euclidean distance for image-to-class assignment. By operating directly in a compressed latent space, *AlignPCA*-2*D* achieves fast, interpretable and accurate classification without sacrificing structural fidelity. We evaluate its performance across multiple publicly available datasets and benchmark it against two leading cryo-EM tools, *RELION* and *Cryo­SPARC*, demonstrating comparable or superior performance with substantially reduced computational cost.

## 2. Methods

The *AlignPCA*-2*D* algorithm follows the classical *k*-means paradigm for unsupervised classification, an approach previously applied to cryo-EM 2D classification by Penczek *et al.* (2015 ▸). The method iteratively alternates between two steps: (i) assigning each particle image to the most similar class representative according to a distance metric (2D alignment) and (ii) updating the class representatives by averaging the images assigned to each class. Repeating these steps progressively improves both the particle assignments and the quality of the class averages.

While the overall workflow resembles the classical *k*-means procedure, *AlignPCA*-2*D* evaluates image similarity in a reduced representation obtained from the Fourier coefficients of the images. Specifically, each particle image is represented by a vector of principal component coefficients computed from its Fourier transform. The PCA basis is computed once and kept fixed during the entire alignment and classification procedure, avoiding iterative recomputation. In this stable latent space, similarity between images is then measured using Euclidean distance in the low-dimensional PCA space, which substantially reduces the dimensionality of the problem while preserving the dataset’s dominant structural variability.

### 2.1. 2D image alignment

Let 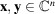 denote the vectorized representations of two images and let *W* be a positive-definite weighting matrix. The weighted quadratic distance between these vectors is defined as
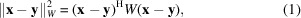
where (·)^H^ denotes the Hermitian transpose.

Let 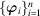 be an orthonormal basis of 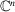 and let 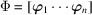 be the unitary matrix whose columns are the basis vectors. Any image can be expanded in this basis as
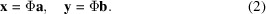


Substituting the expansions (2) into the weighted distance (1) yields 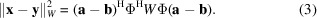


This identity shows that distances can be computed equivalently in any orthogonal representation, including real space, Fourier space or a principal component basis. This property follows from the invariance of inner products under unitary transformations (Parseval’s theorem).

If *W* is diagonal in the chosen basis, the expression in equation (3) simplifies to 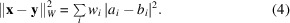


In practical applications of PCA, the representation is often restricted to a limited number of coefficients. For instance, the expansion can be truncated to the first *K* components, ranked in descending order according to their contribution to the total variance, which capture most of the variability in the data: 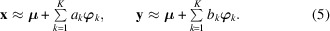


In this case, the distance approximates the full weighted distance, restricted to the retained subspace.

In *AlignPCA*-2*D*, particle images are first transformed into Fourier space and are represented by their Fourier coefficients. In our implementation, the input feature vector for each image is constructed by concatenating the real and imaginary parts of these coefficients. This ensures a linear representation of both phase and amplitude variability within the PCA subspace. PCA is then applied to these vectors, and each image is represented by its projection onto the principal components. Alignment is evaluated in this reduced space by minimizing the Euclidean distance between the PCA coefficient vectors, thereby suppressing noise-dominated components while maintaining high computational efficiency.

In the classification stage, each experimental particle image **y** is compared with the set of class representatives {**x**_*i*_}, where each **x**_*i*_ is the ensemble average of particles currently assigned to class *i*. To control the contribution of different spatial frequencies, a diagonal weighting matrix *W* is introduced, acting as a band-limited pre-emphasis filter. The analysis is restricted to a reliable frequency band, objectively determined by the Fourier ring correlation (FRC), to suppress non­reproducible high-frequency noise. Within this band, progressively larger weights are assigned to higher spatial frequencies, increasing their contribution to the alignment.

The weighting operator *W* is defined in the Fourier domain as a frequency-dependent filter based on the estimated resolution of each class obtained via FRC. Let *f* denote the spatial frequency and *f*_c_ the cutoff frequency corresponding to the resolution at which the FRC drops below a given threshold (*e.g.* 0.143). The filter is defined as the product of a band-limiting low-pass component and a frequency-dependent sharpening term, 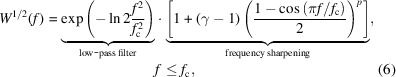
and *W*^1/2^(*f*) = 0 for *f* > *f*_c_.

The Gaussian term ensures a smooth attenuation as the cutoff frequency is approached, while frequencies beyond *f*_c_ are explicitly suppressed to avoid contributions from noise-dominated regions. The sharpening term enhances signal contributions within the reliable frequency band. Here, γ controls the maximum amplification of high frequencies and *p* determines the sharpness of the transition. In practice, *p* is adaptively adjusted as a function of the estimated resolution, following an approximately linear dependence such that higher-resolution class averages receive stronger sharpening. The parameter γ is automatically adjusted at each iteration to control the overall spectral energy after filtering, ensuring stable high-frequency enhancement across different noise conditions.

Because raw experimental particles exhibit very low signal-to-noise ratios, this weighting is applied to the class representatives {**x**_*i*_}, which benefit from the signal averaging of multiple particles. This strategy is equivalent to applying a dampening filter *W*^−1/2^ to the experimental particles, as demonstrated by the following identity: 
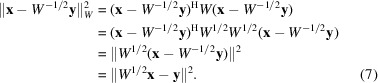


To determine the optimal image-to-class assignment, each representative **x**_*i*_ is transformed according to a set of in-plane parameters **θ** (rotations and translations), generating a set of templates **x**_*i*_(**θ**). The optimal class index i* and the transformation parameters θ* are then obtained by minimizing the weighted Euclidean distance in the Fourier–PCA space: 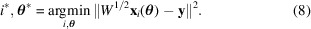


For computational convenience, we define the filtered class representatives as 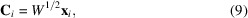
so that the alignment criterion becomes 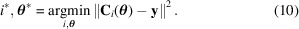


To handle datasets containing millions of particles efficiently, transformations are applied to the class representatives rather than individual images. For each **C**_*i*_, a stack of transformed templates covering an exhaustive grid of rotations and translations is generated. Each particle **y** is then compared against this stack in the reduced PCA space. Once the optimal parameters have been identified, the inverse transformation (θ*)^−1^ is applied to **y** before it is incorporated into the updated class average. The optimal alignment parameters are accumulated and applied across iterations, ensuring a progressive refinement of both the alignment and the class averages while maintaining high computational throughput.

In practice, the continuous transformation parameters **θ** are optimized by means of an exhaustive search over a progressively refined discrete grid using a coarse-to-fine strategy. The angular search initially covers the full range [−180°, 180°] with coarse sampling, while the translational search begins with a window covering up to approximately 12% of the image size, discretized using a coarse step. For a typical 128 × 128 pixel image, this corresponds to an initial angular step of 8° and a translational range of approximately ±15 pixels, with a discretization step size of five pixels along both the *x* and *y* directions. This corresponds to seven discrete shifts along each axis, yielding a total of 49 translational candidates per rotation in the early iterations. As the refinement progresses, both the angular and translational step sizes are successively reduced, reaching 2° and one pixel, respectively, in later stages. This coarse-to-fine scheduling provides a broad capture range during the initial iterations and high-precision alignment at convergence, while keeping the total number of evaluated templates per class representative computationally tractable.

### 2.2. Representative updates

First, a subset of *N*_1_ particles is used to initialize the class averages. Initial references are obtained by applying *k*-means clustering to the PCA coefficient vectors of this subset. This provides a set of initial class prototypes that capture the main structural variability present in the dataset.

**Figure 1:**
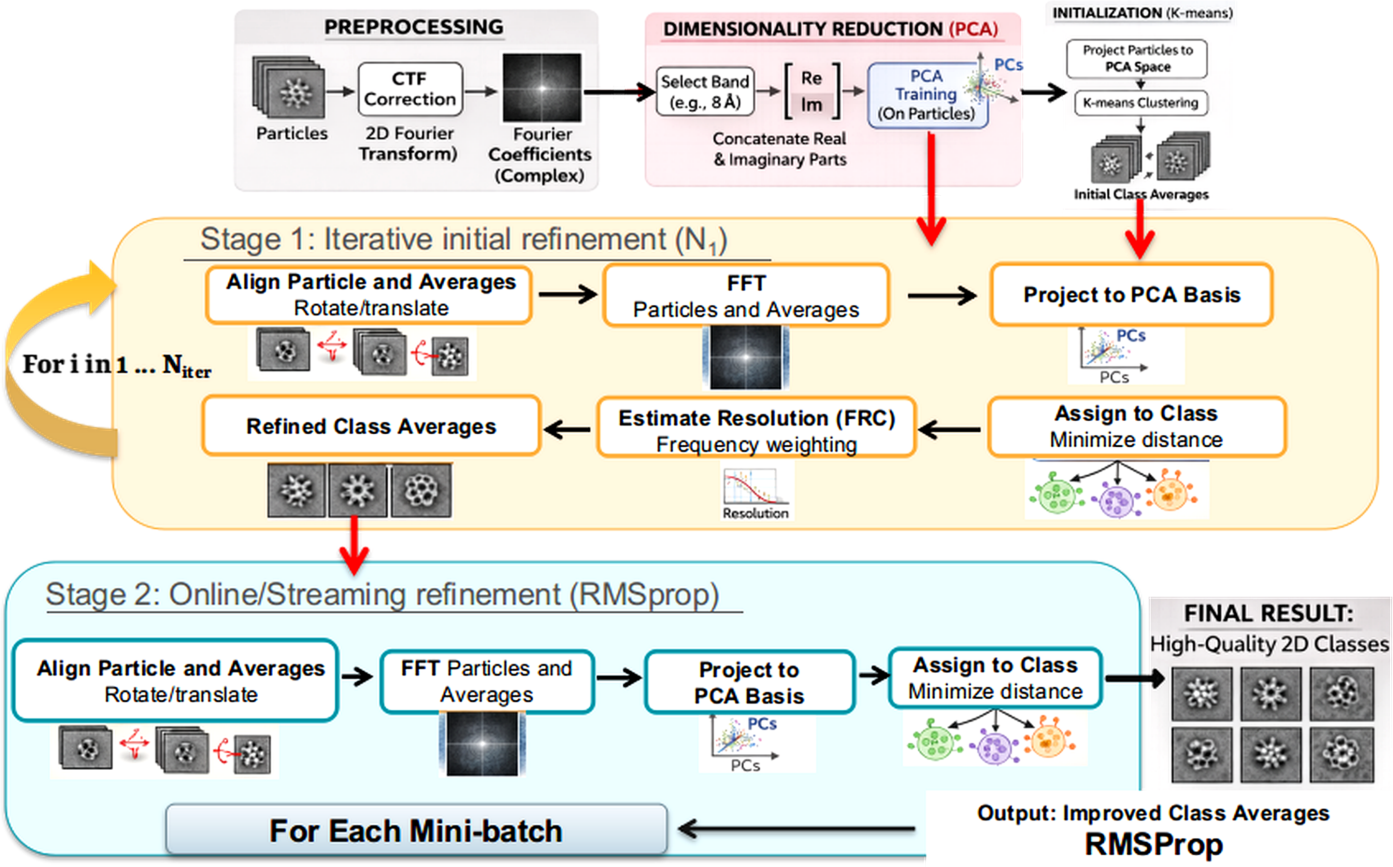
Schematic overview of the *AlignPCA*-2*D* algorithm.

Subsequently, the refinement procedure is carried out in two stages, as shown in Fig. 1 ▸. In Stage 1, class averages are iteratively refined using the subset of *N*_1_ particles. At each iteration, particles are compared with the current class representatives and assigned to the most similar class according to the Euclidean distance between their PCA coefficients derived from the Fourier component representation. The class representatives are then updated by averaging the images assigned to each class, and the resulting averages are filtered by *W*^1/2^ to generate the updated references for the next iteration.

Once the classes stabilize in the first stage, the remaining particles are processed in batches of size *N*_b_ and assigned to their most similar reference classes, again based on the distance in PCA–Fourier space. In the second stage, the class representatives obtained from Stage 1 are further refined using an *RMSProp*-inspired optimization scheme. Let **C**_*t*_ denote the class representation at iteration *t*, and let **g**_*t*_ be the gradient of the cost function, which is defined as the sum of the distances between all images assigned to that class and the current class representation, 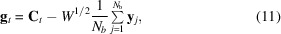
where the sum runs over the *N*_b_ particles in the current batch. The moving average of the squared gradient, **v**_*t*_, is initialized as **v**_0_ = **0** and updated as 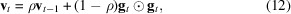
where ρ is the decay factor and ⊙ denotes the Hadamard (element-wise) product. The class representation is subsequently updated according to 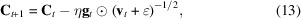
where η is the learning rate and ɛ is a small constant for numerical stability. All operations, including the power and the addition of the constant, are performed element-wise.

This iterative refinement strategy allows the class representations to evolve smoothly as new particle batches are incorporated, yielding stable, progressively more accurate class averages that capture the structural variability in the dataset. The batch updates make our algorithm naturally suited for streaming workflows. As modern cryo-EM facilities achieve faster image acquisition and near real-time data transfer, the ability to process data in parallel with acquisition has become increasingly relevant. Real-time classification enables on-the-fly quality assessment, provides rapid feedback for microscope operators and facilitates the early detection of sample or alignment issues, thereby enhancing the overall efficiency of cryo-EM data collection.

Overall, *AlignPCA*-2*D* combines PCA-based dimensionality reduction in Fourier space, Euclidean-distance alignment in the resulting latent space and iterative class refinement with streaming updates, enabling efficient 2D classification of large cryo-EM datasets. The main steps of the algorithm are summarized in Fig. 1 ▸.

### 2.3. Code availability

*AlignPCA*-2*D* has been developed in Python using PyTorch libraries and is publicly available from *Xmipp* (de la Rosa-Trevín *et al.*, 2013 ▸) in the development branches (which will eventually become the next release of *Xmipp*), and is integrated into the image-processing framework *Scipion* 3 (Conesa *et al.*, 2023 ▸).

## 3. Results

To evaluate the performance of *AlignPCA*-2*D*, we first conducted a controlled validation using a simulated dataset. To assess its robustness under diverse experimental conditions, we further analyzed several publicly available cryo-EM datasets from EMPIAR. The results were compared with those obtained using the widely adopted tools *RELION* v.5 and *CryoSPARC* v.4.6. *RELION* employs a variable-metric gradient-descent (VDAM) algorithm for 2D classification, while *CryoSPARC* uses stochastic gradient-based optimization to maximize the data likelihood. All datasets were processed within the *Scipion* 3 framework to ensure reproducibility and consistent data handling across methods.

For the experimental datasets, aligned micrographs were imported into *Scipion*, and CTF parameters were estimated using both *Gctf* (Zhang, 2016 ▸) and *CTFFind*4 (Rohou & Grigorieff, 2015 ▸). A consensus of CTF parameters was then generated using *Xmipp* (Sorzano *et al.*, 2021 ▸), retaining only micrographs with an estimated resolution better than 7 Å. Particle picking was automatically performed with *crYOLO* (Wagner *et al.*, 2019 ▸), followed by extraction and downstream 2D classification using the respective algorithms under comparison. The extracted particle box size was set to 128 × 128 pixels in all cases to maintain consistency across datasets and methods. For *CryoSPARC* and *RELION*, default classification parameters were used to ensure a fair and reproducible comparison.

In our approach, CTF correction was applied using the Wiener filter implementation in *Xmipp* before PCA projection and classification. Only Fourier coefficients corresponding to spatial frequencies lower than 8 Å were retained, as they contain the dominant alignment-relevant signal (Scheres, 2012 ▸). This value was chosen as a practical default that provided stable alignment across the datasets analyzed in this study. As the optimal frequency range for alignment may depend on the structural characteristics of the particle, the *Scipion* implementation of *AlignPCA*-2*D* provides this cutoff as a user-adjustable parameter. For all datasets, the number of PCA components retained in *AlignPCA*-2*D* corresponded to those that captured 75% of the total variance, ensuring a compact yet informative latent representation. Although this variance threshold was fixed, the actual number of components varied according to the intrinsic variability of each dataset. Particle selection for all three methods was performed via manual inspection of 2D class averages. Particles belonging to classes with suboptimal structural features or high noise levels were excluded, following standard cryo-EM data-curation protocols.

### 3.1. Dataset analysis

#### 3.1.1. Simulated dataset

To evaluate the accuracy of *AlignPCA*-2*D*, we conducted a controlled study using a simulated dataset generated within the *Scipion* 3 framework. A 3D volume of *Escherichia coli* β-galactosidase bound to PETG (Bartesaghi *et al.*, 2015 ▸) was used as the ground-truth model to generate ten distinct 2D projections. These projections, with a box size of 128 × 128 pixels, represent different viewing directions of the macromolecule (Supplementary Fig. S1*a*).

To mimic experimental conditions, each projection was replicated 3000 times, resulting in a dataset of 30 000 particle images grouped by projection angle in sequential blocks. Each particle was subjected to random in-plane rotations and translations within ±15 pixels (Supplementary Fig. S1*c*). The contrast transfer function (CTF) was simulated using parameters representative of high-end cryo-electron microscopes: an accelerating voltage of 300 kV, a spherical aberration (*C*_s_) of 2.7 mm and an amplitude contrast (*Q*_0_) of 0.07. Defocus values were randomly sampled between 0.6 and 2.0 µm (Supplementary Fig. S1*d*). Finally, additive white Gaussian noise was applied to achieve a signal-to-noise ratio (SNR) of 0.01 (Supplementary Fig. S1*e*).

**Figure 2:**
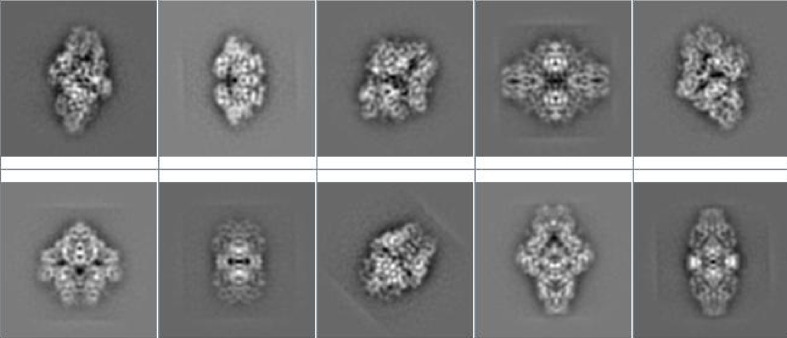
Representative 2D class averages from the simulated dataset generated by *AlignPCA*-2*D*.

The dataset was processed using *AlignPCA*-2*D*, *Cryo­SPARC* and *RELION*, with the number of classes set to 50 for all methods (Fig. 2 ▸ and Supplementary Figs. S2–S4). Classification accuracy was quantified by tracking the ground-truth projection index for each particle, identifying outliers as particles assigned to classes inconsistent with their original projection.

*AlignPCA*-2*D* isolated 830 particles into ‘garbage’ classes (the final classes in Supplementary Fig. S2), which lacked discernible structural features. Among the remaining 29 170 particles assigned to structurally meaningful classes, only 44 were misclassified. These errors were primarily observed in smaller, lower-resolution classes.

In comparison, although *CryoSPARC* and *RELION* produced visually well defined averages in most classes, quantitative analysis of particle-distribution histograms (Supplementary Figs. S3*b* and S4*b*) indicated that both methods frequently grouped particles from distinct projections within the same class. This heterogeneity was reflected in more blurred class averages, consistent with suboptimal alignment of particle subsets. By contrast, *AlignPCA*-2*D* separated these subsets into more homogeneous classes, reducing the impact of mismatched particles on the resulting averages.

To balance classification accuracy with computational efficiency, we evaluated cumulative PCA variance thresholds ranging from 55% to 95% (Supplementary Table S1). Lower thresholds reduced the execution times by retaining fewer components but slightly increased the number of rejected particles (from 770 at 95% to 1367 at 55%). Despite these differences, the fraction of discarded particles remained low and had a negligible effect on class-average quality. Based on these results, a threshold of 75% was chosen as the default, providing an optimal compromise between structural detail, classification precision and computational efficiency.

#### 3.1.2. EMPIAR-10061

The first experimental dataset, EMPIAR-10061 (Bartesaghi *et al.*, 2015 ▸), contains raw cryo-EM images of β-galactosidase, a canonical benchmark in structural biology. A total of 1539 micrographs were processed in *Scipion* (see Section 2), yielding 381 792 automatically extracted particles used for 2D classification, with the number of classes fixed at 100 in all cases. For this dataset, 1416 principal components were retained, representing 47.2% of the real and imaginary Fourier coefficients and accounting for 75% of the variance in the data.

**Figure 3:**
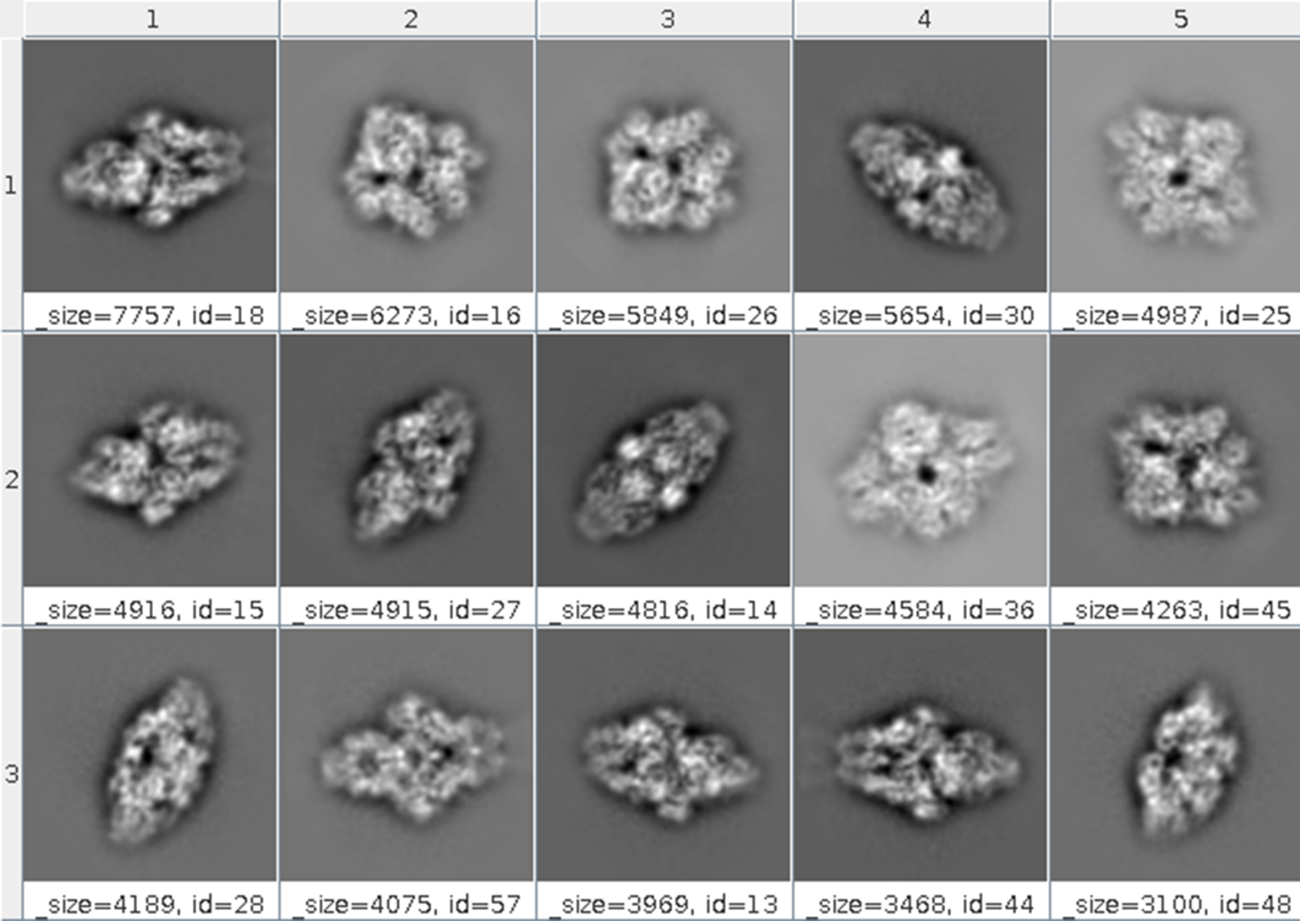
Representative 2D class averages from the experimental EMPIAR-10061 dataset obtained with *AlignPCA*-2*D*.

Fig. 3 ▸ and Supplementary Fig. S9 show the class averages obtained using *AlignPCA*-2*D*, whereas Supplementary Fig. S5 shows a comparison of the classes obtained using *Cryo­SPARC*, *RELION* and *AlignPCA*-2*D*. Additionally, Table 1 ▸ summarizes the computation times required for each method to generate 100 classes. When processing 381 792 particles, *RELION* requires more than four hours, whereas *Cryo­SPARC* and *AlignPCA*-2*D* completed the task in 30.11 and 29.33 min, respectively.

Supplementary Fig. S5 illustrates that all three methods successfully separated distinct particle orientations, while Supplementary Fig. S9 demonstrates that *AlignPCA*-2*D* effectively discriminates signal from noise. Table 2 ▸ summarizes the number of particles retained after classification and the number of particles commonly identified between methods. Manual class selection was applied consistently across all approaches, retaining only classes that exhibited well defined structural features and minimal noise. A total of 119 435 particles were selected by *RELION*, 132 408 by *CryoSPARC* and 132 923 by *AlignPCA*-2*D*. The number of commonly identified particles was computed using the consensus tools available in *Scipion* (Sorzano *et al.*, 2021 ▸). The highest agreement was observed between *CryoSPARC* and *AlignPCA*-2*D*, with 80.8% of the total particles (132 923). The agreement between *RELION* and *CryoSPARC* reached 78.6%, while the proportion of shared particles between *RELION* and *AlignPCA*-2*D* was 73.4%.

**Figure 4:**
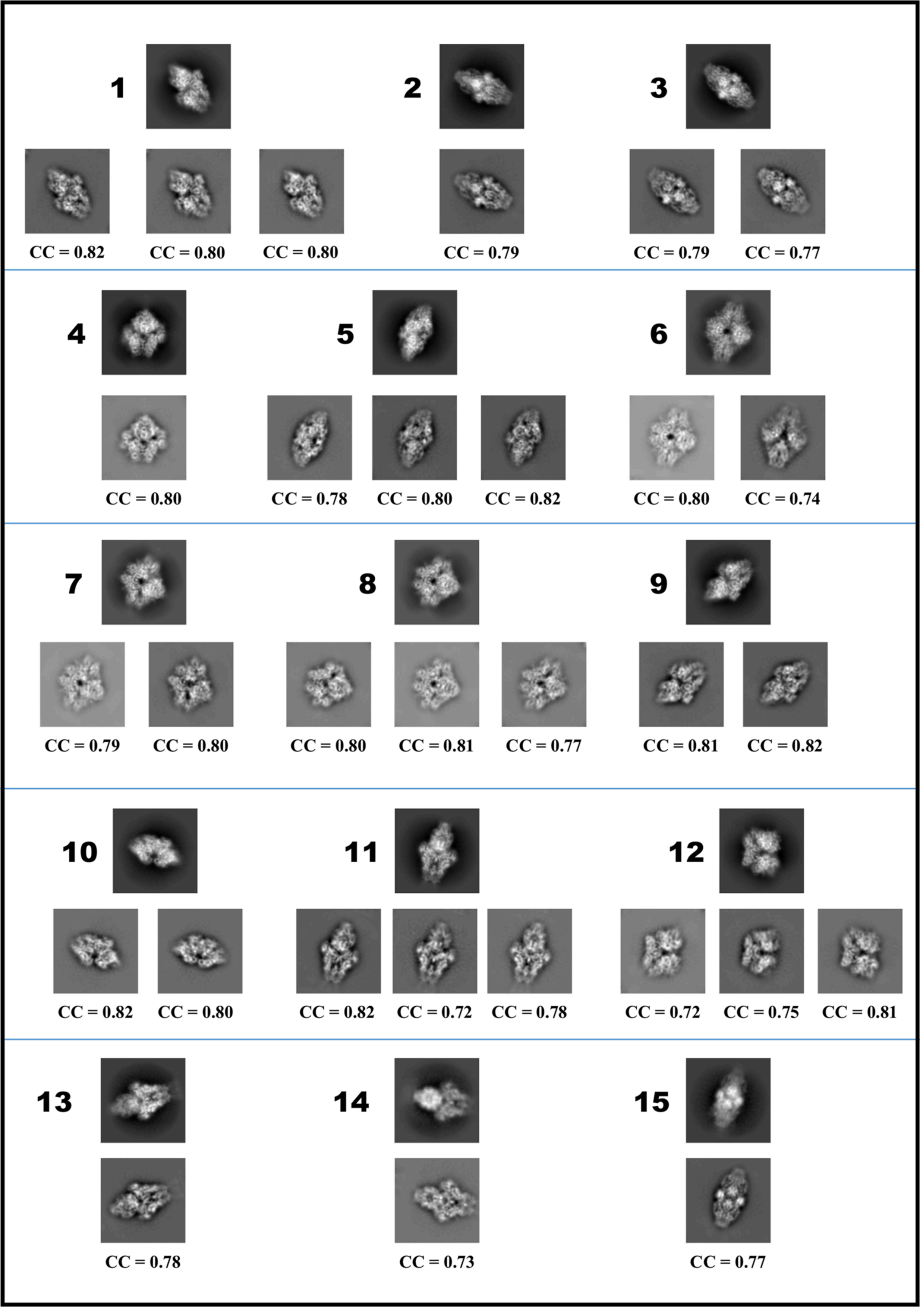
Comparison of class averages from *AlignPCA*-2*D* and *CryoSPARC*. *AlignPCA*-2*D* averages (bottom rows) were aligned with their corresponding *CryoSPARC* templates (top rows), and similarity was quantified using cross-correlation (CC). *AlignPCA*-2*D* identified a larger number of distinct averages, with multiple classes corresponding to a single *CryoSPARC* class, suggesting a greater ability to resolve subtle structural variations.

To examine structural consistency between methods, we compared class averages obtained with *AlignPCA*-2*D* against those generated by *CryoSPARC* and *RELION*. After manual curation of the 100 initial classes, 30 high-quality classes were retained for *AlignPCA*-2*D*, 15 for *CryoSPARC* and 29 for *RELION*. The correspondence between methods is illustrated in Fig. 4 ▸ and Supplementary Fig. S12.

*AlignPCA*-2*D* class averages were aligned with those from *CryoSPARC* and *RELION*, and similarity was quantified using cross-correlation (CC). The results indicate that the 15 *CryoSPARC* classes are well represented within the *AlignPCA*-2*D* classification, with CC values around 0.80 (Fig. 4 ▸). In several cases, a single *CryoSPARC* class corresponds to multiple *AlignPCA*-2*D* classes, suggesting that the PCA-based approach partitions the data into finer structural states.

**Figure 5:**
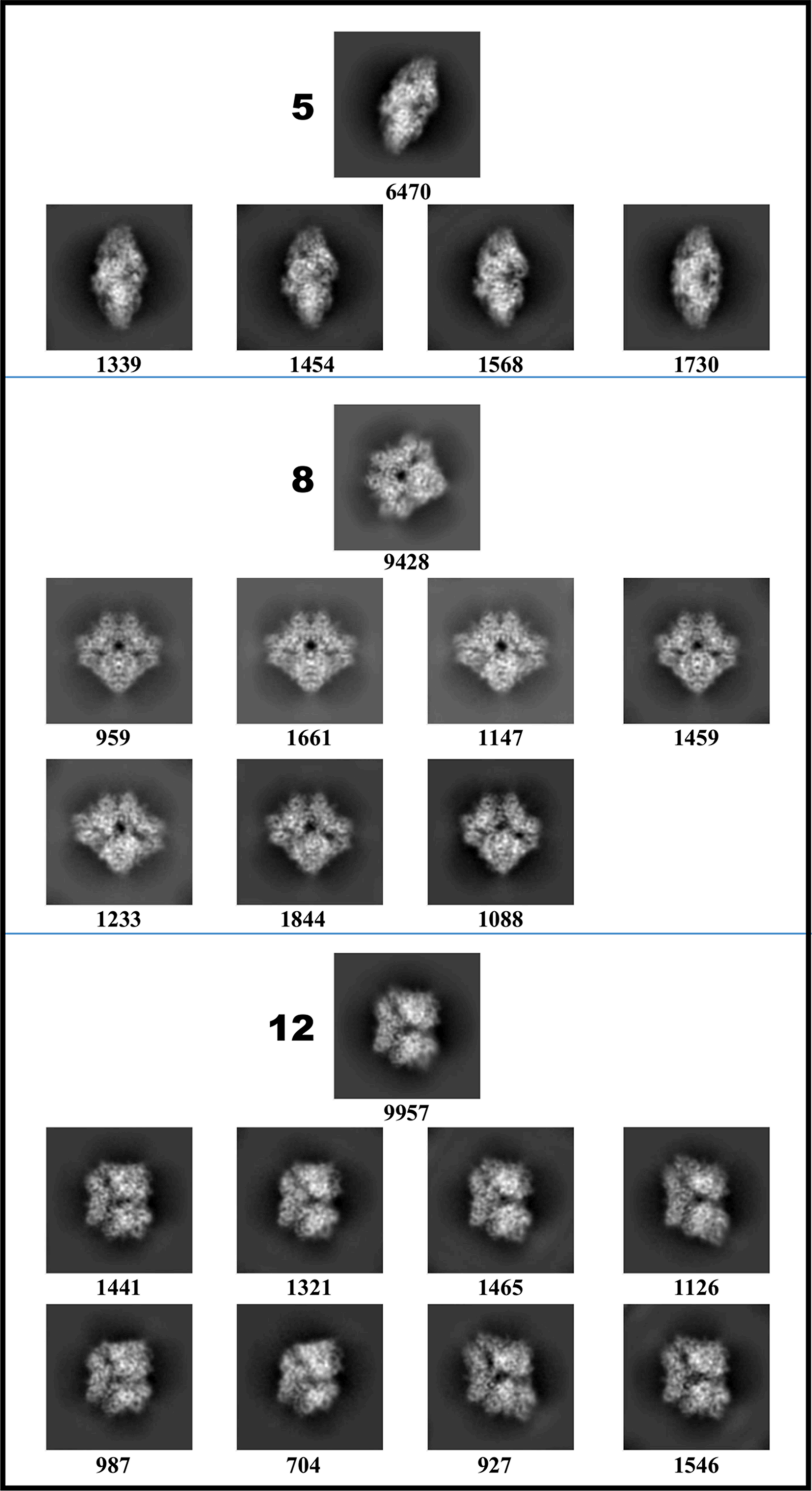
Subclassification of heterogeneous *CryoSPARC* classes. A second round of 2D classification was performed in *CryoSPARC* on particles from classes 5, 8 and 12 (top), selected from those shown in Fig. 4 ▸. The resulting subclasses (bottom) reveal that these averages contain multiple distinct projection orientations that were previously grouped together. Particle numbers for each subclass are indicated below. These observations may reflect latent structural heterogeneity within standard 2D averages.

To further assess this effect, we performed a subclassification analysis on selected *CryoSPARC* classes (classes 5, 8 and 12). These apparently homogeneous classes decomposed into multiple distinct projection views upon reclassification (Fig. 5 ▸), which are consistent with those identified by *AlignPCA*-2*D* in a single pass.

So far, our analysis has focused on manually selected subsets of well defined classes, which leaves little room for ambiguity. Since this represents an early stage of the classification pipeline, where a relatively permissive selection is typically applied before a more stringent second round, it is expected that some classes may contain a small proportion of noisy or poorly aligned particles.

To further evaluate the particle-selection behavior of our method, we analyzed a subset of *CryoSPARC* classes (68 classes) that lacked well defined structural features (*i.e.* ‘noise classes’), comprising 212 838 particles (Supplementary Fig. S13). This set of particles was reprocessed using *CryoSPARC*, *RELION* and *AlignPCA*-2*D*, and the resulting class averages are shown in Supplementary Figs. S14, S15 and S16, respectively. As observed, neither *CryoSPARC* nor *RELION* produced any classes with well defined structural features. In contrast, *AlignPCA*-2*D* successfully generated coherent classes containing approximately 23 707 well aligned particles, corresponding to about 11.14% of the total dataset.

To assess the robustness of the alignments determined by *AlignPCA*-2*D*, we applied the orientations obtained by our method to the same particle subset. We re-ran both *Cryo­SPARC* and *RELION* using these alignment parameters as initial estimates. Under these conditions, *CryoSPARC* successfully aligned approximately 37 800 particles, producing interpretable class averages (Supplementary Fig. S17), while *RELION* generated high-quality classes comprising roughly 39 300 particles (Supplementary Fig. S18). A detailed inspection revealed that the majority of the particles recovered under these conditions were significantly displaced from the image center.

These results demonstrate that despite comparable overall computation times, *AlignPCA*-2*D* provides a more exhaustive exploration of the rotational and translational spaces, enabling the recovery of particles that would otherwise be discarded or misaligned by *CryoSPARC*. Moreover, the reduced dependence of *AlignPCA*-2*D* on the initial centering accuracy of particle extractions makes it less sensitive to imperfections introduced during particle picking. Consequently, smaller extraction boxes can be employed without compromising classification quality, thereby reducing computational cost.

Importantly, by re-centering particles based on the alignment parameters obtained with *AlignPCA*-2*D*, particles initially rejected by other methods can be effectively reintegrated into downstream analyses. Together, these findings highlight that *AlignPCA*-2*D* enhances classification completeness and particle retention, providing a computationally efficient and robust alternative for the early stages of cryo-EM data processing.

#### 3.1.3. EMPIAR-10025

**Figure 6:**
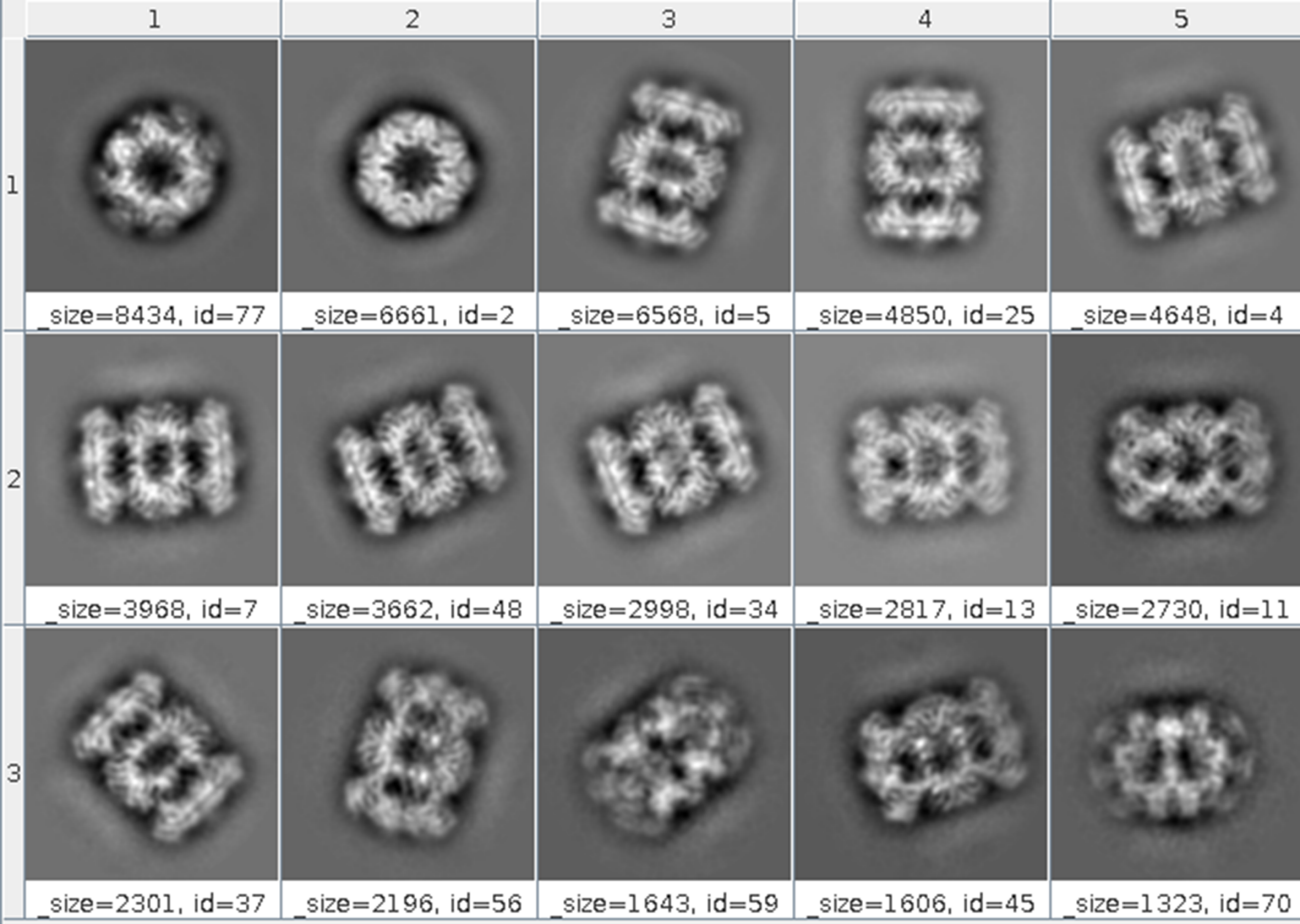
Representative 2D class averages from the EMPIAR-10025 dataset obtained with *AlignPCA*-2*D*.

The second experimental dataset tested, EMPIAR-10025 (Campbell *et al.*, 2015 ▸), consists of cryo-EM images of the T20S proteasome. A total of 195 micrographs were processed in *Scipion*, yielding 161 358 extracted particles. For this dataset, 1231 principal components were retained, representing 38.4% of the total number of Fourier coefficients. Fig. 6 ▸ and Supplementary Fig. S6 illustrate representative classes obtained using the different methods, while Supplementary Fig. S10 shows the 100 classes obtained with *AlignPCA*-2*D*. The computation times, shown in Table 1 ▸, were comparable for *CryoSPARC* and *AlignPCA*-2*D* (17.48 and 14.47 min, respectively), whereas *RELION* was significantly slower, requiring 95.49 min.

The number of particles selected after classification was 136 773 for *RELION*, 144 809 for *CryoSPARC* and 144 891 for *AlignPCA*-2*D*. Table 2 ▸ summarizes these results, along with the number of particles consistently identified across methods, which was close to 90% in all cases. On this dataset, *AlignPCA*-2*D* matches *CryoSPARC* in both classification quality and particle-selection consistency, while maintaining a faster or at least comparable computational performance.

#### 3.1.4. EMPIAR-10081: HCN1 ion channel

The third experimental dataset, EMPIAR-10081 (Lee & MacKinnon, 2017 ▸), corresponds to a membrane protein (HCN1 ion channel), which typically poses greater challenges due to structural flexibility and heterogeneity. After pre­processing, 258 426 particles were extracted. For this dataset, 2476 principal components were retained, representing 44.9% of the Fourier coefficients.

**Figure 7:**
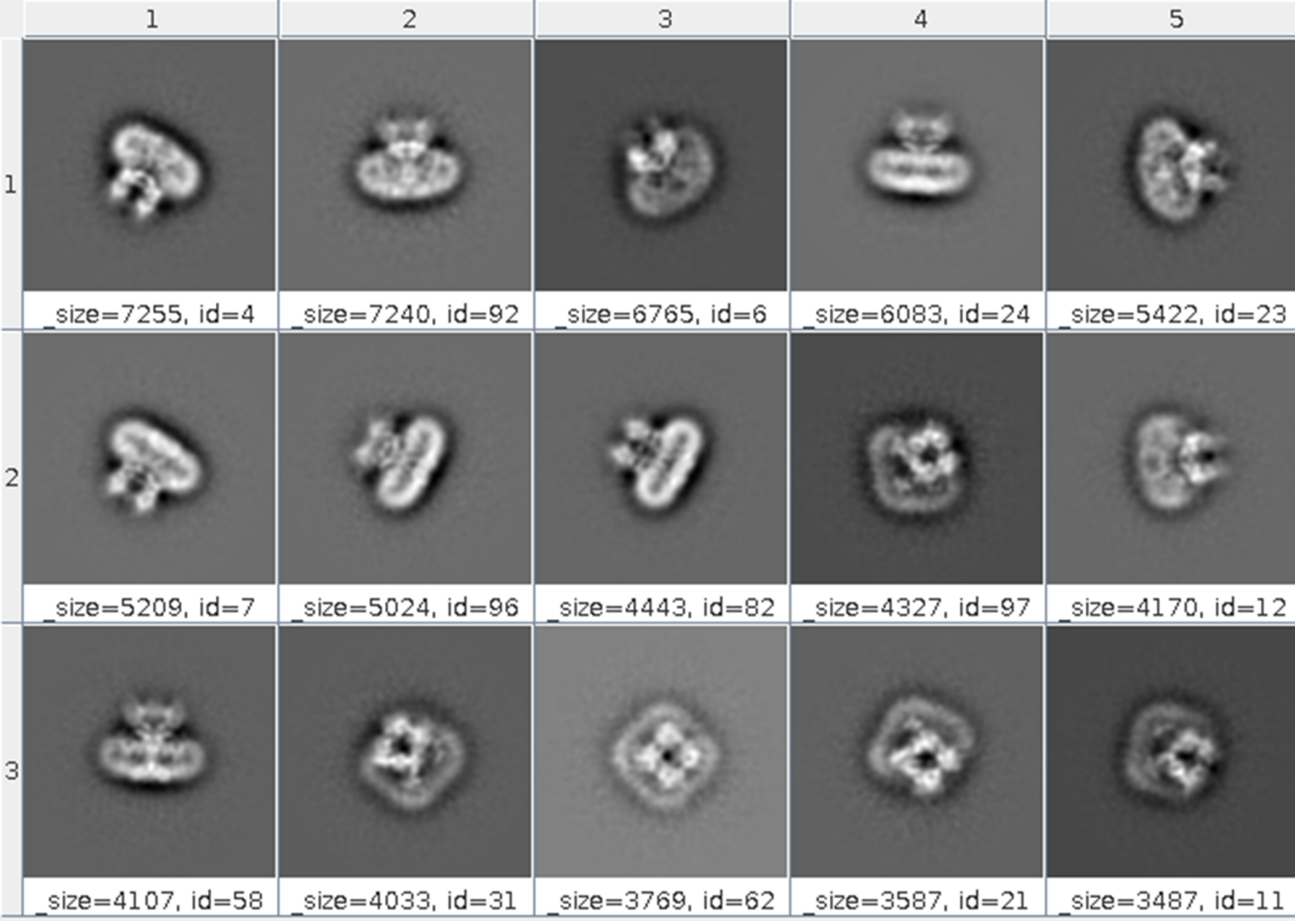
Representative 2D class averages from the EMPIAR-10081 dataset obtained with *AlignPCA*-2*D*.

The 2D classification performed with *RELION* required nearly four hours, whereas *CryoSPARC* and *AlignPCA*-2*D* completed the task in 34 and 30 min, respectively. All methods successfully distinguished different particle orientations and produced high-quality class averages (Fig. 7 ▸ and Supplementary Fig. S7). The consensus analysis revealed a high degree of particle consistency, with approximately 80% concordance among the three methods (Table 2 ▸).

Even for this high-complexity membrane-protein dataset, *AlignPCA*-2*D* demonstrated robust classification performance (Supplementary Fig. S11), generating well defined class averages while maintaining computation times comparable to *CryoSPARC* and significantly faster than *RELION*.

#### 3.1.5. EMPIAR-11604

The fourth experimental dataset corresponds to the AP2 clathrin adaptor complex in the presence of heparin (Partlow *et al.*, 2022 ▸). A total of 3046 cryo-EM micrographs were preprocessed, resulting in the extraction of 1 107 902 particles for subsequent classification. This large-scale dataset is particularly well suited for evaluating the scalability of *AlignPCA*-2*D*. For this dataset, 2D classification was performed into 400 classes. In this case, 1209 principal components were retained, representing 46.2% of the total number of Fourier coefficients.

**Figure 8:**
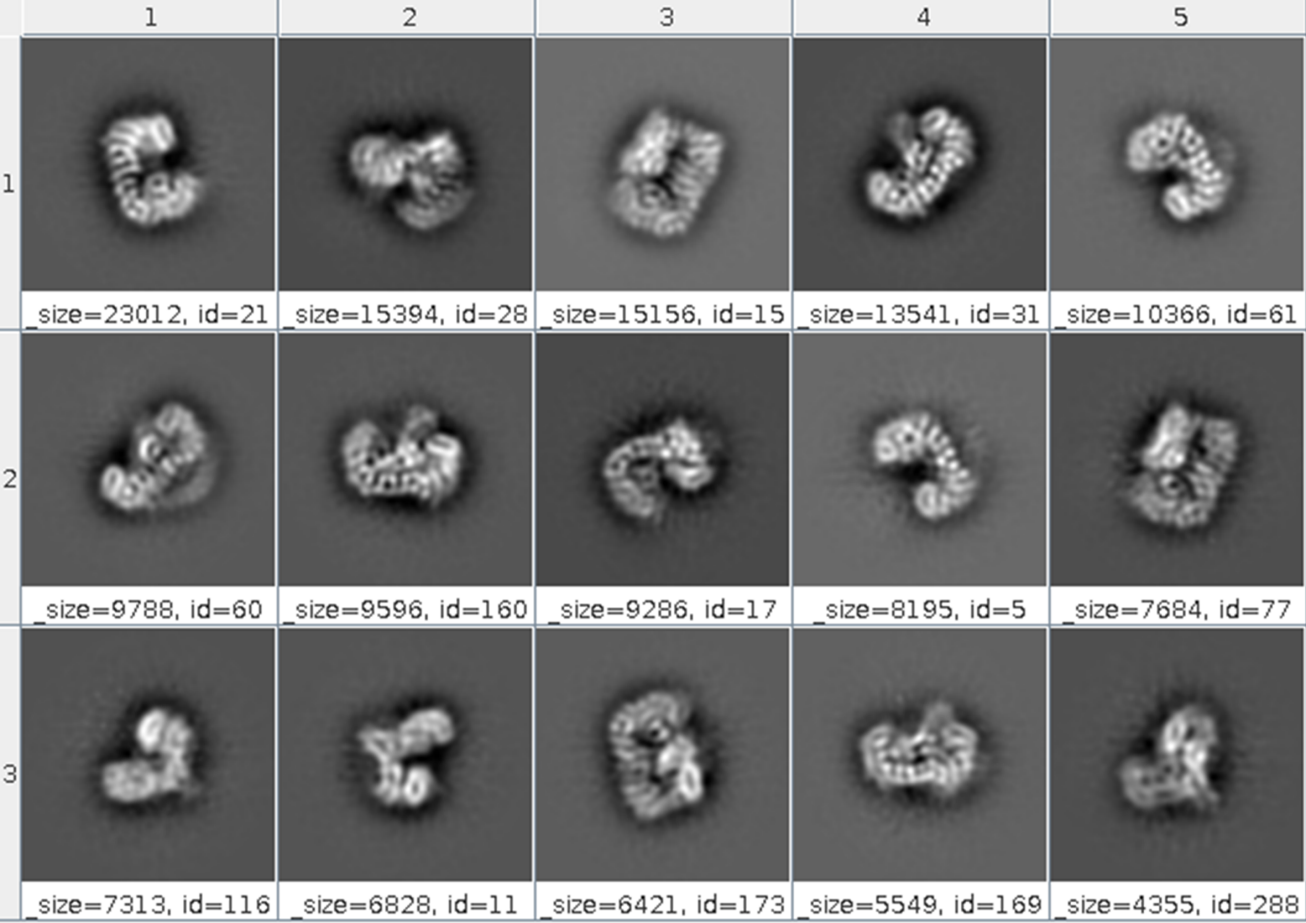
Representative 2D class averages from the EMPIAR-11604 dataset obtained with *AlignPCA*-2*D*.

Fig. 8 ▸ and Supplementary Fig. S8 show representative class averages obtained with the different methods, in which distinct particle orientations are clearly observed in all cases. Table 1 ▸ summarizes the computation times: *CryoSPARC* required 206 min (∼3.4 h) and *RELION* required 2220 min (37 h), whereas *AlignPCA*-2*D* completed the classification in only 122min (∼2 h).

Regarding particle consistency, Table 2 ▸ shows the agreement between the methods using our consensus approach. In this analysis, approximately 60% of the selected particles were consistently identified across the platforms. This level of agreement reflects the distinct underlying algorithms and search strategies employed by each method. Despite these variations in particle selection, the class averages remain consistent, highlighting the complementary nature of these algorithmic approaches in capturing the structural diversity of the dataset. Overall, these results demonstrate that *AlignPCA*-2*D* achieves robust classification performance while maintaining excellent scalability, enabling efficient processing of large-scale cryo-EM datasets.

In the presented cases, we observed that all three tested methods successfully separated particles into distinct classes corresponding to different macromolecular orientations (Supplementary Figs. S5–S8). This demonstrates the robustness of our PCA-based algorithm for 2D classification. Additionally, *AlignPCA*-2*D* processing times were comparable to those of *CryoSPARC* and significantly faster than those of *RELION*. The highly competitive computation times achieved further highlight the efficiency of our method without compromising classification accuracy. Such efficiency is crucial for high-throughput cryo-EM workflows, where time savings can greatly enhance overall processing pipelines.

### 3.2. *AlignPCA*-2*D* in streaming cryo-EM workflows

Traditional 2D classification in cryo-EM is typically performed as a static process that is initiated once all particles have been collected. However, modern high-throughput data acquisition makes it increasingly desirable to perform classification in a streaming manner, allowing real-time assessment of data quality and sample heterogeneity. To support real-time data analysis during cryo-EM acquisition, *AlignPCA*-2*D* was integrated into the *Scipion* framework for fully streaming operation. In this mode, an initial subset of particles is used to generate PCA-based reference classes. As new particles are acquired, they are automatically projected into the pre-learned PCA space and assigned to their most similar classes using Euclidean distance. Class averages are continuously refined through batch updates following the *RMSProp* scheme.

This implementation enables near-real-time monitoring of sample quality and orientation diversity, allowing users to detect issues such as mispicking or preferred orientations during acquisition.

## 4. Discussion

Dimensionality reduction has long been used in cryo-EM as a strategy to manage the high variability and large volume of particle datasets. In this work, we demonstrate that principal component analysis (PCA) can be used not only as a data-compression technique but also as an effective method for aligning and classifying data. While grounded in the same linear dimensionality-reduction principles that underpin classical MSA approaches, *AlignPCA*-2*D* reformulates the problem as a Fourier-based latent-space framework for scalable and streaming 2D classification. In this framework, alignment is handled explicitly, which allows the PCA basis to remain fixed throughout the process, avoiding the iterative recomputation typical of classical methods.

Results across multiple EMPIAR datasets show that *AlignPCA*-2*D* performs comparably to state-of-the-art tools such as *RELION* and *CryoSPARC*, while offering faster execution and a fully open-source implementation. Beyond its computational efficiency, *AlignPCA*-2*D* introduces a conceptual shift: alignment and classification in a reduced, well defined latent space can be sufficient to capture the essential structural variability of cryo-EM data. Its integration within *Scipion* and compatibility with streaming acquisition further extend its applicability, enabling real-time classification and adaptive feedback during data collection.

*AlignPCA*-2*D* achieves its performance through a set of methodological choices that make the algorithm particularly well suited for modern large-scale cryo-EM datasets. Particle images are represented through a PCA compression of their Fourier coefficients, which reduces the dimensionality of the problem while preserving the dominant structural variability of the data. Both particle images and class representatives are embedded in a shared low-dimensional latent space, enabling alignment and classification to be performed using simple Euclidean distance calculations. A frequency-dependent spectral weighting scheme emphasizes reproducible structural information while suppressing noise-dominated components, improving the robustness of the alignment process. In addition, class representatives are updated through an incremental optimization strategy based on *RMSProp*, allowing stable and efficient updates from large streams of particle assignments and enabling the method to scale efficiently to very large datasets. Together, these design choices transform the traditionally expensive 2D classification problem into an efficient optimization problem in a compact latent space.

Furthermore, while this implementation prioritizes computational simplicity, several extensions of PCA specifically tailored to cryo-EM data have been proposed, including rotationally invariant representations (Zhao & Singer, 2014 ▸) and CTF-aware covariance-estimation approaches (Marshall *et al.*, 2023 ▸). These approaches have been shown to improve the quality of 2D classification in cryo-EM datasets. Although these variants were not explored in the present work, their integration within the *AlignPCA*-2*D* framework could be investigated in future work.

The open availability of the method promotes transparency, reproducibility and community-driven development, which are critical factors for advancing high-throughput cryo-EM workflows. Together, these properties position *AlignPCA*-2*D* as a lightweight, transparent and accessible alternative for 2D classification, combining speed, interpretability and open science.

## Supplementary Material

Supplementary Table and Figures. DOI: 10.1107/S2059798326004572/vo5022sup1.pdf

## Figures and Tables

**Table 1:** Comparison of computation times for different 2D classification methods

EMPIAR	No. of particles	*CryoSPARC* (min)	*RELION* (min)	*AlignPCA*-2*D* (min)
10061	381792	30.11	248.33	29.33
10025	181358	20.45	95.49	16.00
10081	258426	34.00	237.50	30.40
11604	1107902	206.00	2220.00	122.00

**Table 2:** Analysis of particle retention and the number of particles commonly identified across different 2D classification methods Percentages relative to the maximum number of particles among the compared methods are given in parentheses. Bold values indicate the highest percentage of commonly identified particles for a given structure.

EMPIAR	*RELION*	*CryoSPARC*	*AlignPCA*-2*D*	*CryoSPARC*–*RELION* (%)	*CryoSPARC*–*AlignPCA*-2*D* (%)	*RELION*–*AlignPCA*-2*D* (%)
10061	119435	132408	132923	104050 (78.6)	**108149 (82.7)**	97618 (73.4)
10025	136773	144809	144891	**130152 (89.9)**	**130232 (89.9)**	125967 (86.9)
10081	123494	125921	127963	102747 (81.6)	**108497 (84.8)**	100913 (78.9)
11604	405267	332065	332679	235419 (58.1)	**209545 (63.0)**	213144 (52.6)

## References

[bb1] Bartesaghi, A., Merk, A., Banerjee, S., Matthies, D., Wu, X., Milne, J. L. S. & Subramaniam, S. (2015). *Science*, **348**, 1147–1151. 10.1126/science.aab1576 PMC6512338 25953817

[bb2] Bhamre, T., Zhao, Z. & Singer, A. (2017). *Proc. IEEE Int. Symp. Biomed. Imaging*, **2017**, 654–658 10.1109/ISBI.2017.7950605 PMC5657188 29081898

[bb3] Campbell, M. G., Veesler, D., Cheng, A., Potter, C. S. & Carragher, B. (2015). *eLife*, **4**, e06380. 10.7554/eLife.06380 PMC4391500 25760083

[bb4] Conesa, P., Fonseca, Y. C., de la Morena, J. J., Sharov, G., de la Rosa-Trevín, J. M., Cuervo, A., Mena, A. G., de Francisco, B. R., del Hoyo, D., Herreros, D., Marchan, D., Strelak, D., Fernández-Giménez, E., Ramírez-Aportela, E., de Isidro-Gómez, F. P., Sánchez, I., Krieger, J., Vilas, J. L., del Caño, L., Gragera, M., Iceta, M., Martínez, M., Losana, P., Melero, R., Marabini, R., Carazo, J. M. & Sorzano, C. O. S. (2023). *Biol. Imaging*, **3**, e13.

[bb5] de la Rosa-Trevín, J. M., Otón, J., Marabini, R., Zaldívar, A., Vargas, J., Carazo, J. M. & Sorzano, C. O. S. (2013). *J. Struct. Biol.* **184**, 321–328. 10.1016/j.jsb.2013.09.015 24075951

[bb6] Frank, J., Radermacher, M., Penczek, P., Zhu, J., Li, Y., Ladjadj, M. & Leith, A. (1996). *J. Struct. Biol.* **116**, 190–199. 10.1006/jsbi.1996.0030 8742743

[bb7] Lee, C.-H. & MacKinnon, R. (2017). *Cell*, **168**, 111–120. 10.1016/j.cell.2016.12.023 PMC5496774 28086084

[bb8] Marshall, N. F., Mickelin, O., Shi, Y. & Singer, A. (2023). *Biol. Imaging*, **3**, e2. 10.1017/S2633903X23000028 PMC10465116 37645688

[bb9] Partlow, E. A., Cannon, K. S., Hollopeter, G. & Baker, R. W. (2022). *Nat. Struct. Mol. Biol.* **29**, 339–347. 10.1038/s41594-022-00749-z PMC10116491 35347313

[bb10] Penczek, P., Zhu, J. & Frank, J. (2015). *Ultramicroscopy*, **63**, 205–218. 10.1016/0304-3991(96)00037-x 8921628

[bb11] Punjani, A., Rubinstein, J. L., Fleet, D. J. & Brubaker, M. A. (2017). *Nat. Methods*, **14**, 290–296. 10.1038/nmeth.4169 28165473

[bb12] Rao, R., Moscovich, A. & Singer, A. (2020). *arXiv*:2010.09989.

[bb13] Rohou, A. & Grigorieff, N. (2015). *J. Struct. Biol.* **192**, 216–221. 10.1016/j.jsb.2015.08.008 PMC6760662 26278980

[bb14] Scheres, S. H. W. (2012). *J. Struct. Biol.* **180**, 519–530. 10.1016/j.jsb.2012.09.006 PMC3690530 23000701

[bb15] Sorzano, C. O. S., Jiménez-Moreno, A., Maluenda, D., Ramírez-Aportela, E., Martínez, M., Cuervo, A., Melero, R., Conesa, J. J., Sánchez-García, R., Strelak, D., Fernández-Giménez, E., de Isidro-Gómez, F., Herreros, D., Conesa, P., del Caño, L., Fonseca, Y., Jiménez de la Morena, J., Macías, J. R., Losana, P. & Carazo, J.-M. (2021). *Methods Mol. Biol.* **2305**, 257–289.

[bb16] van Heel, M. & Frank, J. (1981). *Ultramicroscopy*, **6**, 187–194. 10.1016/0304-3991(81)90059-0 7268930

[bb17] van Heel, M., Harauz, G., Orlova, E. V., Schmidt, R. & Schatz, M. (1996). *J. Struct. Biol.* **116**, 17–24. 10.1006/jsbi.1996.0004 8742718

[bb18] Wagner, T., Merino, F., Stabrin, M., Moriya, T., Antoni, C., Apelbaum, A., Hagel, P., Sitsel, O., Raisch, T., Prumbaum, D., Quentin, D., Roderer, D., Tacke, S., Siebolds, B., Schubert, E., Shaikh, T. R., Lill, P., Gatsogiannis, C. & Raunser, S. (2019). *Commun. Biol.* **2**, 218. 10.1038/s42003-019-0437-z PMC6584505 31240256

[bb19] Zhang, K. (2016). *J. Struct. Biol.* **193**, 1–12. 10.1016/j.jsb.2015.11.003 PMC4711343 26592709

[bb20] Zhao, Z. & Singer, A. (2014). *J. Struct. Biol.* **186**, 153–166. 10.1016/j.jsb.2014.03.003 PMC4014198 24631969

