# Supplementary material for: AlignPCA-2D: PCA-reduced Euclidean vector alignment for 2D classification in cryo-EM
Source: Acta Crystallogr D Struct Biol. 2026 Jun 8;82(Pt 7):727–39. doi: 10.1107/S2059798326004572 (PMC13317688; doi:10.1107/S2059798326004572)
Supplement: Supplementary file 1 [file d-82-00727-sup1.pdf]

STRUCTURAL BIOLOGY

Volume 82 (2026)

Supporting information for article:

AlignPCA-2D: PCA-reduced Euclidean vector alignment for 2D classification in cryo-EM

E. Ramírez-Aportela, O. Zarrabeitia, Y. Fonseca, T. Ceska, S. Subramaniam, J. M. Carazo and C. O. S. Sorzano

Supplementary Table S1

Supplementary Table S1: Performance analysis across different PCA variance thresholds.

**PCA % Variance**	55 %	65 %	75 %	85 %	95 %
**Number of PCs (*K*)**	916	1177	1472	1815	2240
**Discarded Particles**	1,367	974	830	764	770
**Execution Time (min)**	3.39	3.55	4.20	5.03	5.55

The table summarizes the impact of the cumulative variance threshold on the number of retained principal components (K), particle classification (discarded particles), and computational efficiency (execution time) for the simulated dataset (30,000 particles).

Supplementary Figure S1

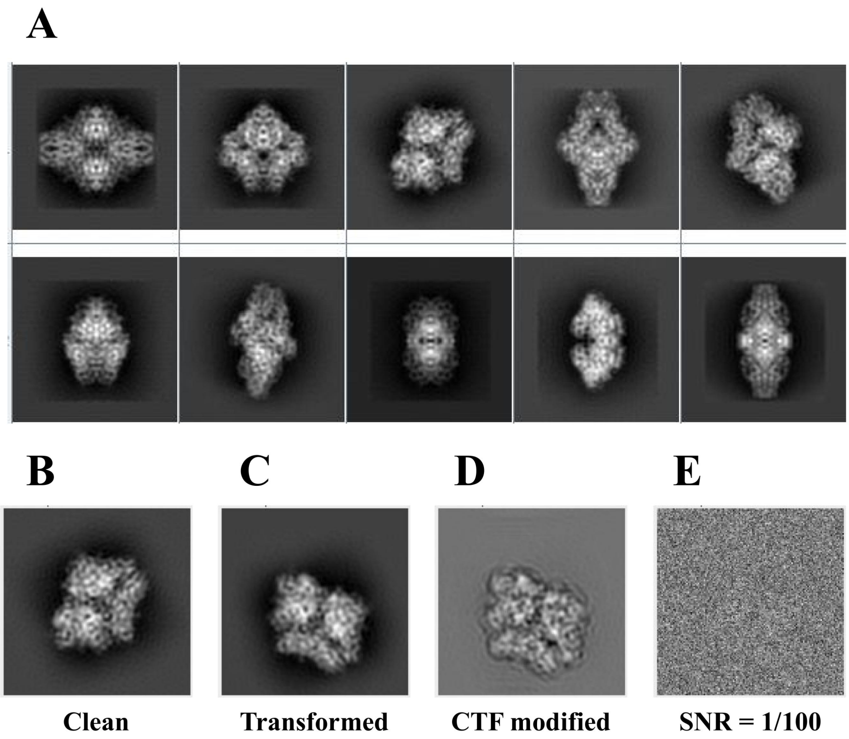

Supplementary Figure S1: Simulated β-galactosidase projection images. (A) Gallery of 10 different clean (noise-free) 2D projections. (B) Simulated clean, centered projection image. (C) The same projection subjected to a random geometric transformation (rotation and translation). (D) Clean, "Transformed" image modified by Contrast Transfer Function. (E) Final particle image containing the transformed, CTF-modified projection contaminated with additive white Gaussian noise at SNR=1/100.

Supplementary Figure S2

Simulated data - AlignPCA-2D

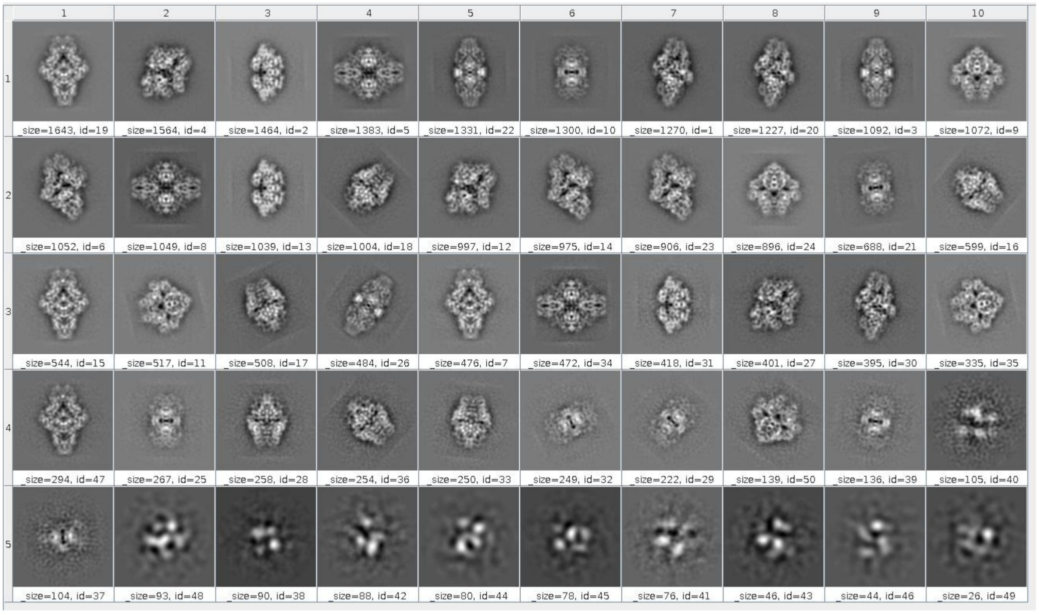

Supplementary Figure S2: Final 2D class averages of the simulated β-galactosidase dataset obtained using AlignPCA-2D.

Supplementary Figure S3

A

Simulated data - CryoSPARC

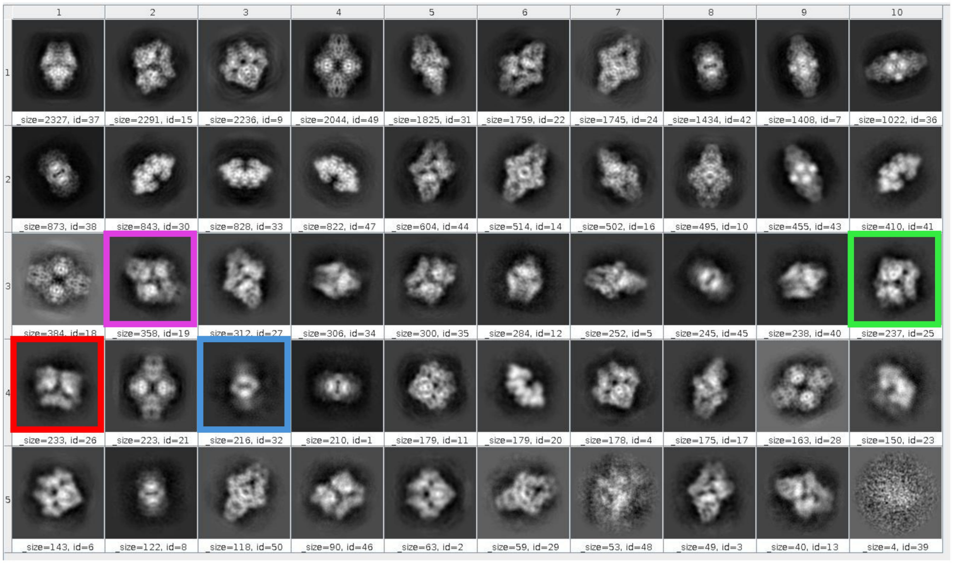

B

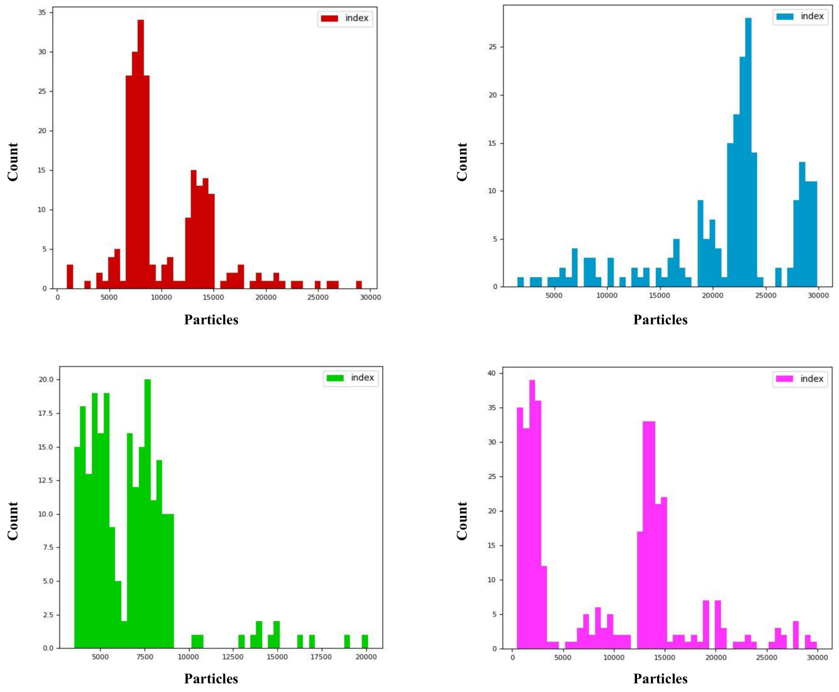

Supplementary Figure S3: Purity analysis of 2D class averages in cryoSPARC. (A) Final 2D class averages of the simulated *E. coli* β-galactosidase dataset obtained using cryoSPARC. Boxes highlight representative classes selected for the detailed purity analysis shown in (B). (B) Particle distribution histograms for the selected classes. The presence of multiple peaks in the histograms indicates non-homogeneous particle populations.

Supplementary Figure sS4

A

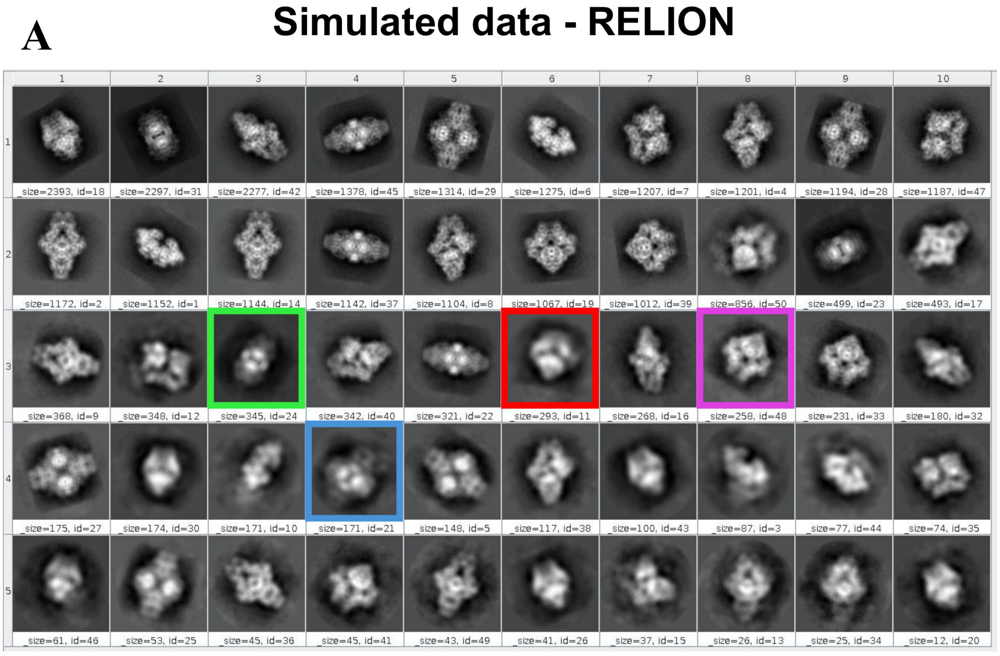

B

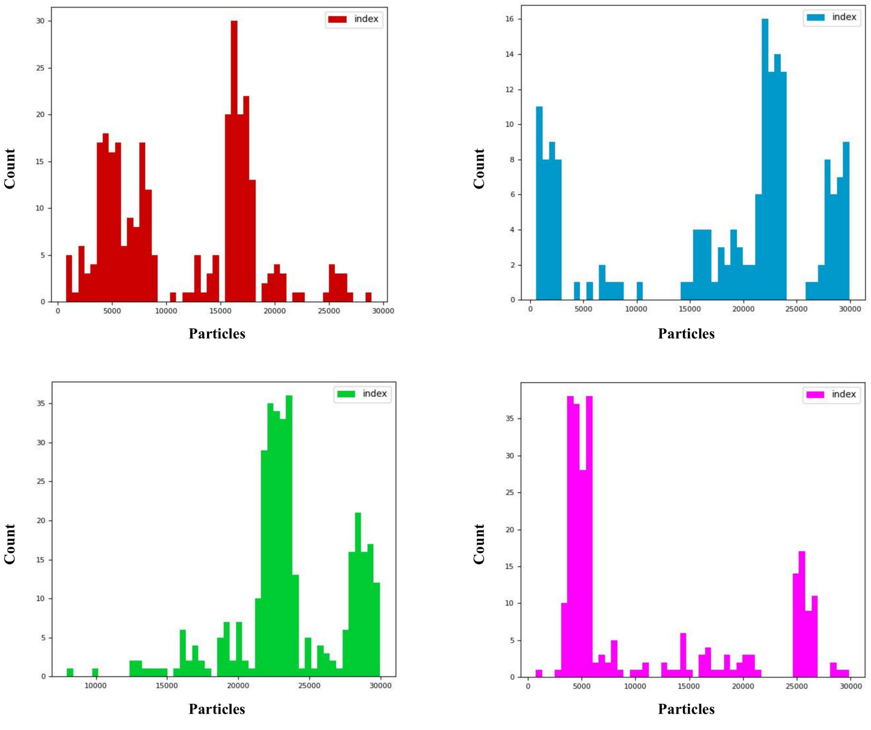

Supplementary Figure S4: Purity analysis of 2D class averages in RELION. (A) Final 2D class averages of the simulated *E. coli* β-galactosidase dataset obtained using RELION. Boxes highlight representative classes selected for the detailed purity analysis shown in (B). (B) Particle distribution histograms for the selected classes. The presence of multiple peaks in the histograms indicates non-homogeneous particle populations.

Supplementary Figure S5

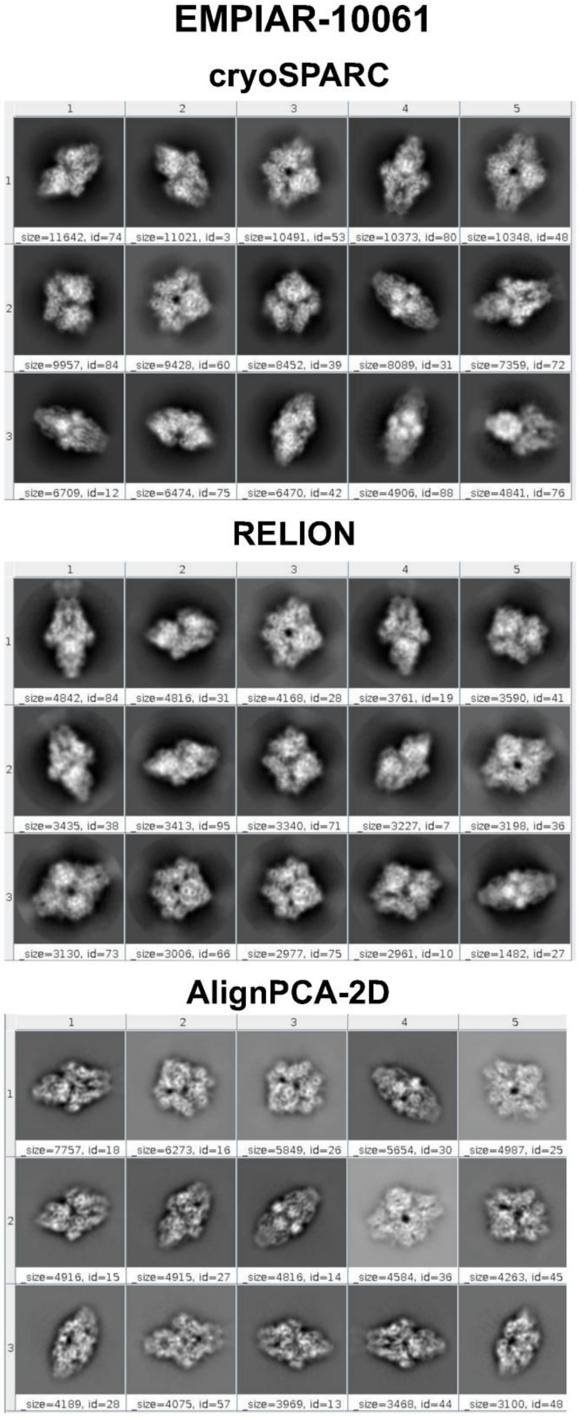

Supplementary Figure S5: Comparison of representative 2D class averages from the EMPIAR-10061 dataset obtained using CryoSPARC, RELION and AlignPCA-2D.

Supplementary Figure S6

EMPIAR-10025

cryoSPARC

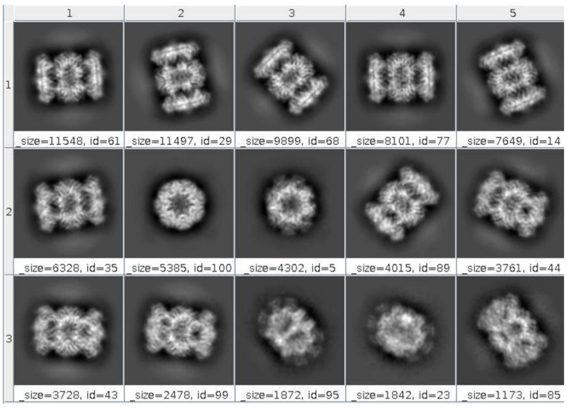

RELION

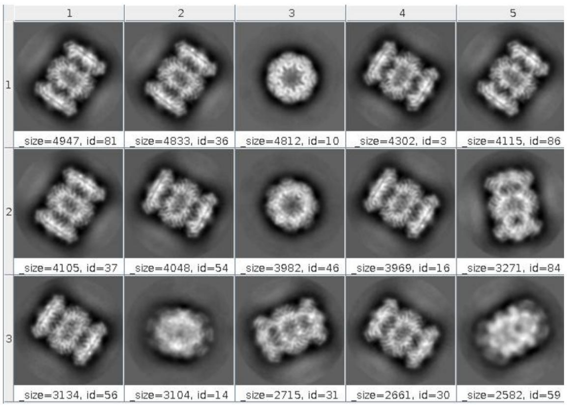

AlignPCA-2D

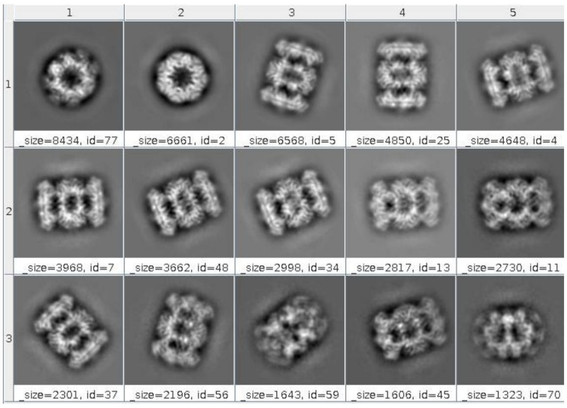

Supplementary Figure S6: Comparison of representative 2D class averages from the EMPIAR-10025 dataset obtained using CryoSPARC, RELION and AlignPCA-2D.

Supplementary Figure S7

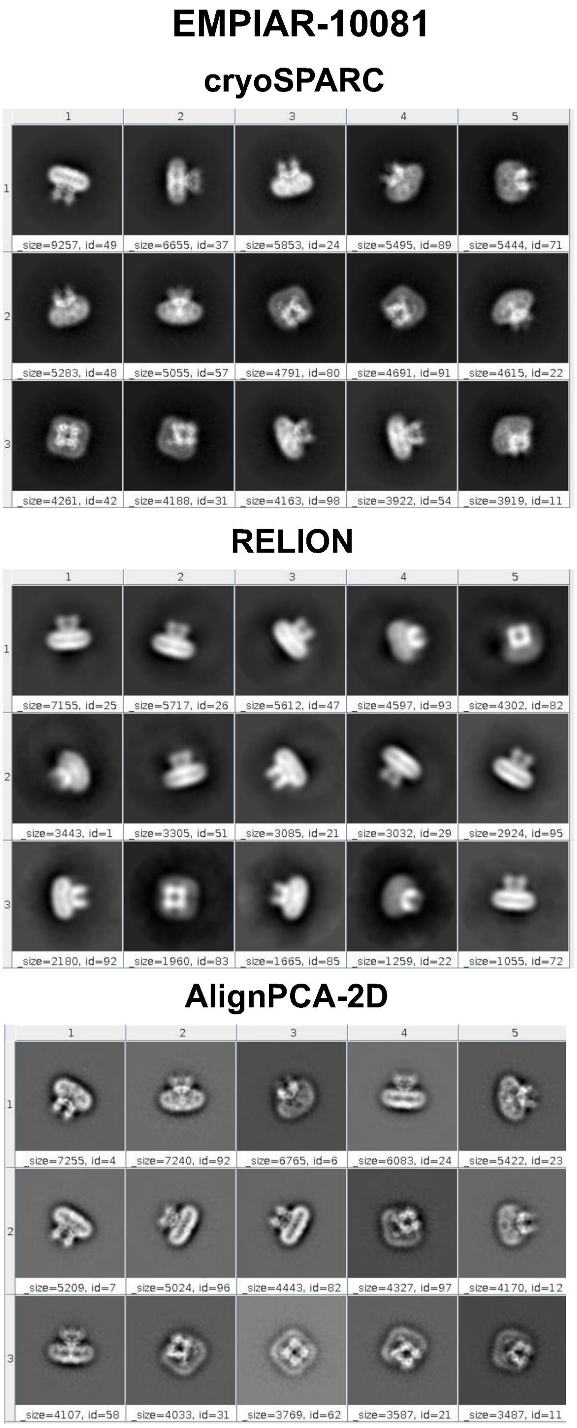

Supplementary Figure S7: Comparison of representative 2D class averages from the EMPIAR-10081 dataset obtained using CryoSPARC, RELION and AlignPCA-2D.

Supplementary Figure S8

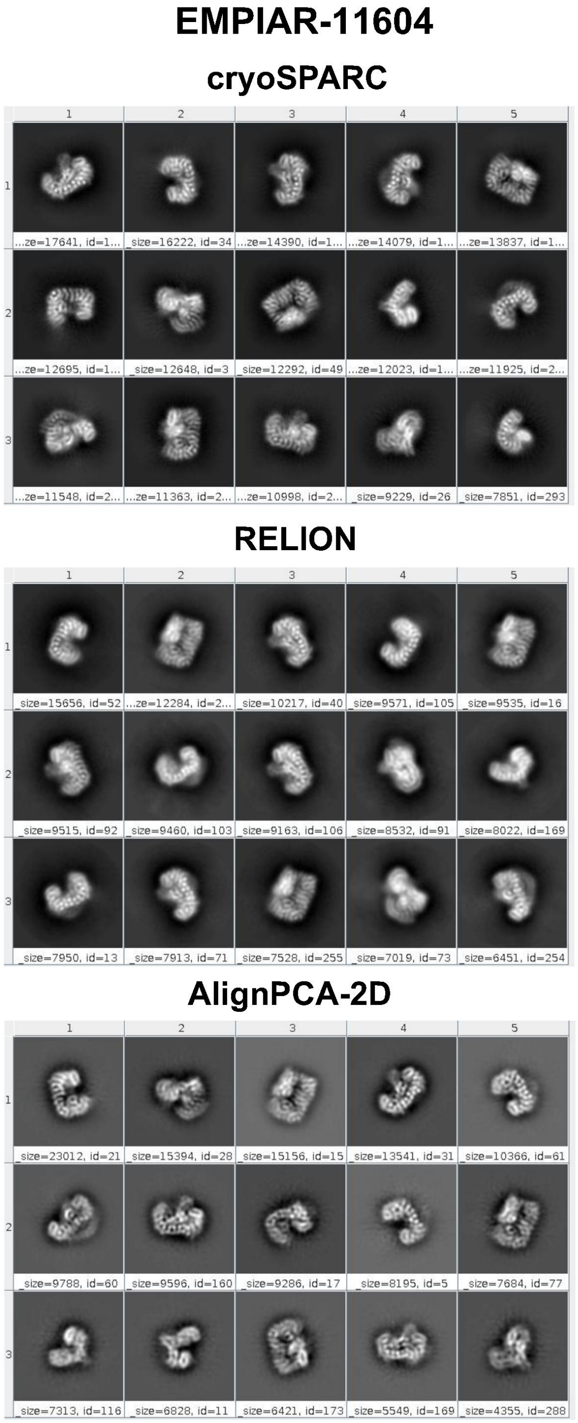

Supplementary Figure S8: Comparison of representative 2D class averages from the EMPIAR-11604 dataset obtained using CryoSPARC, RELION and AlignPCA-2D.

Supplementary Figure S9

EMPIAR-10061

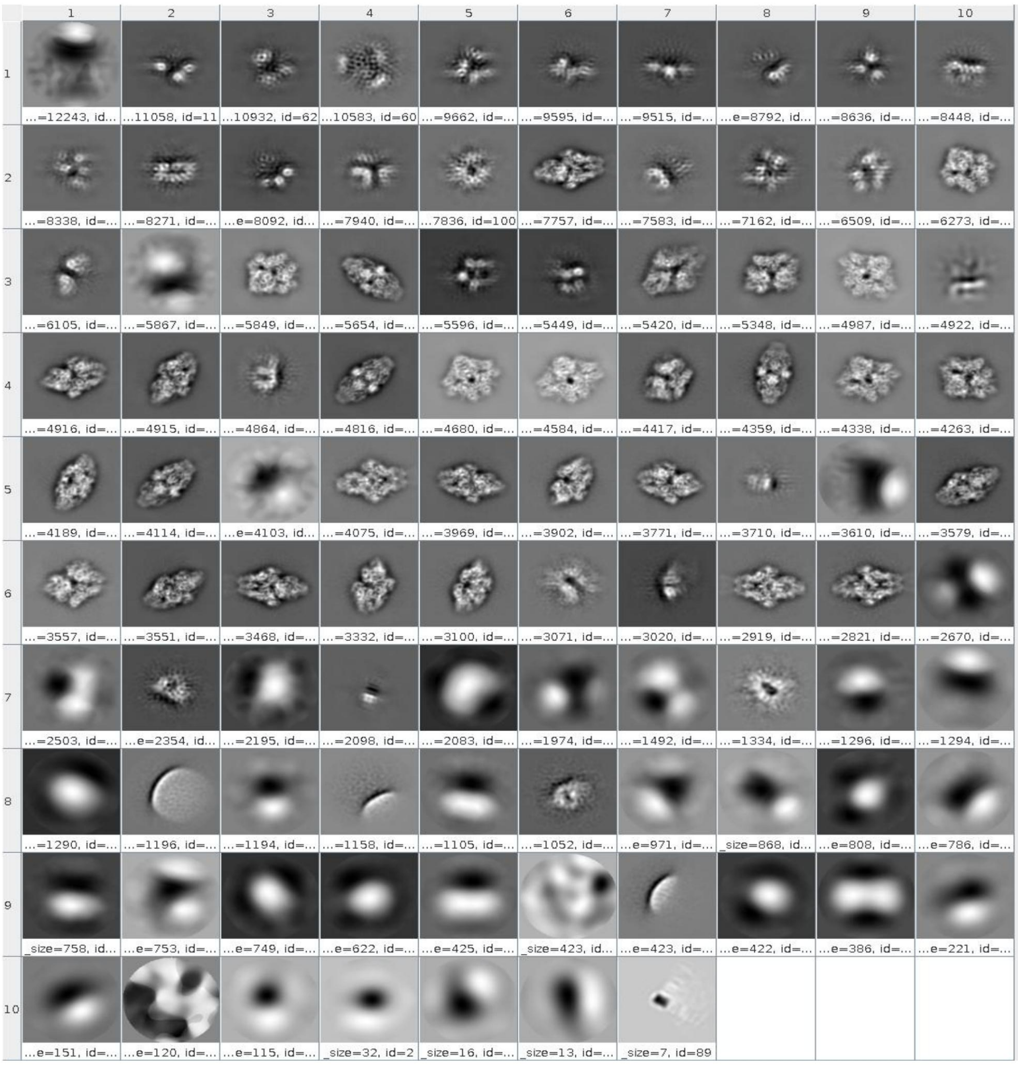

Supplementary Figure S9: Final 2D class averages from the EMPIAR-10061 dataset obtained using AlignPCA-2D.

Supplementary Figure S10

EMPIAR-10025

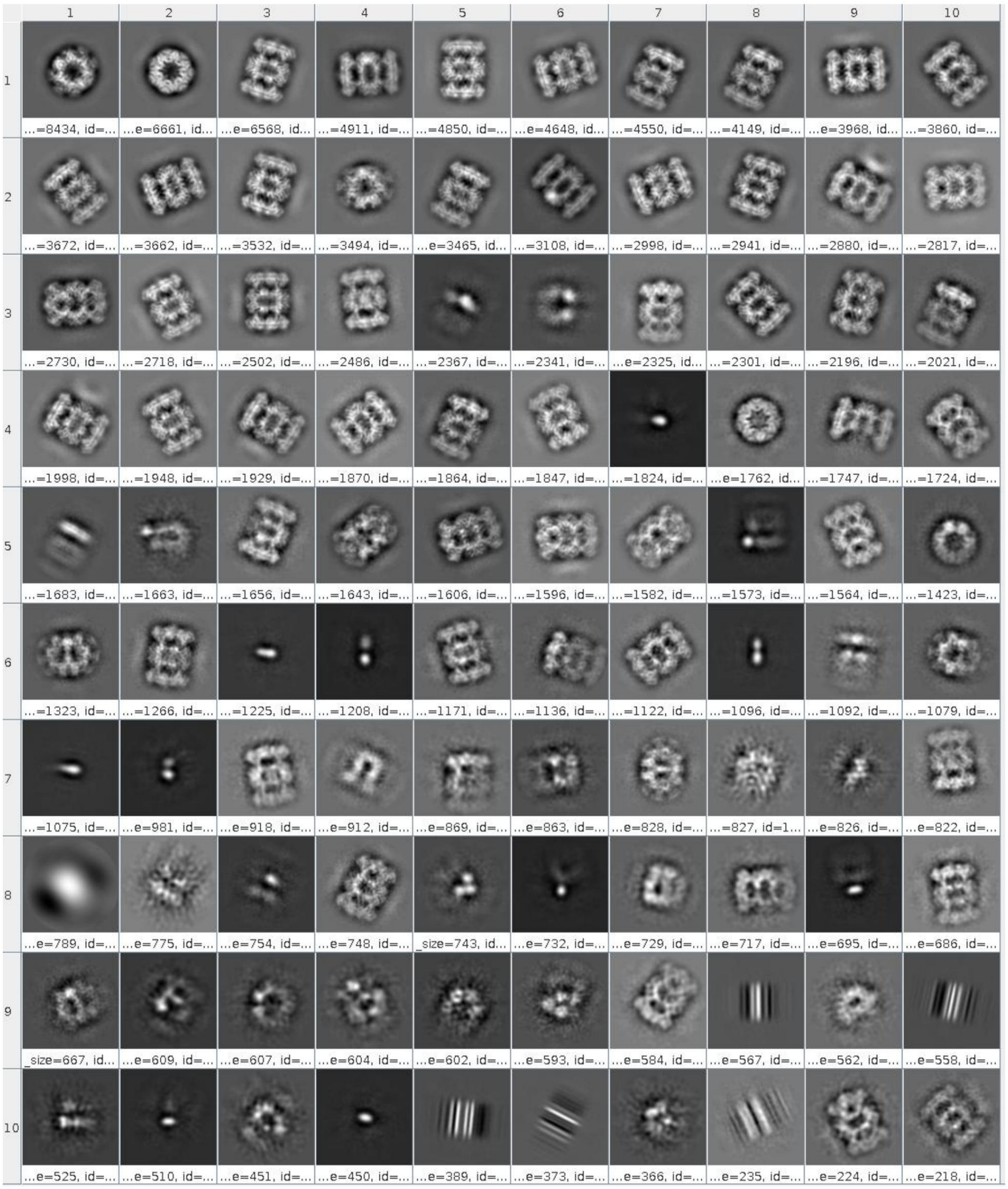

Supplementary Figure S10: Final 2D class averages from the EMPIAR-10025 dataset obtained using AlignPCA-2D.

Supplementary Figure S11

EMPIAR-10081

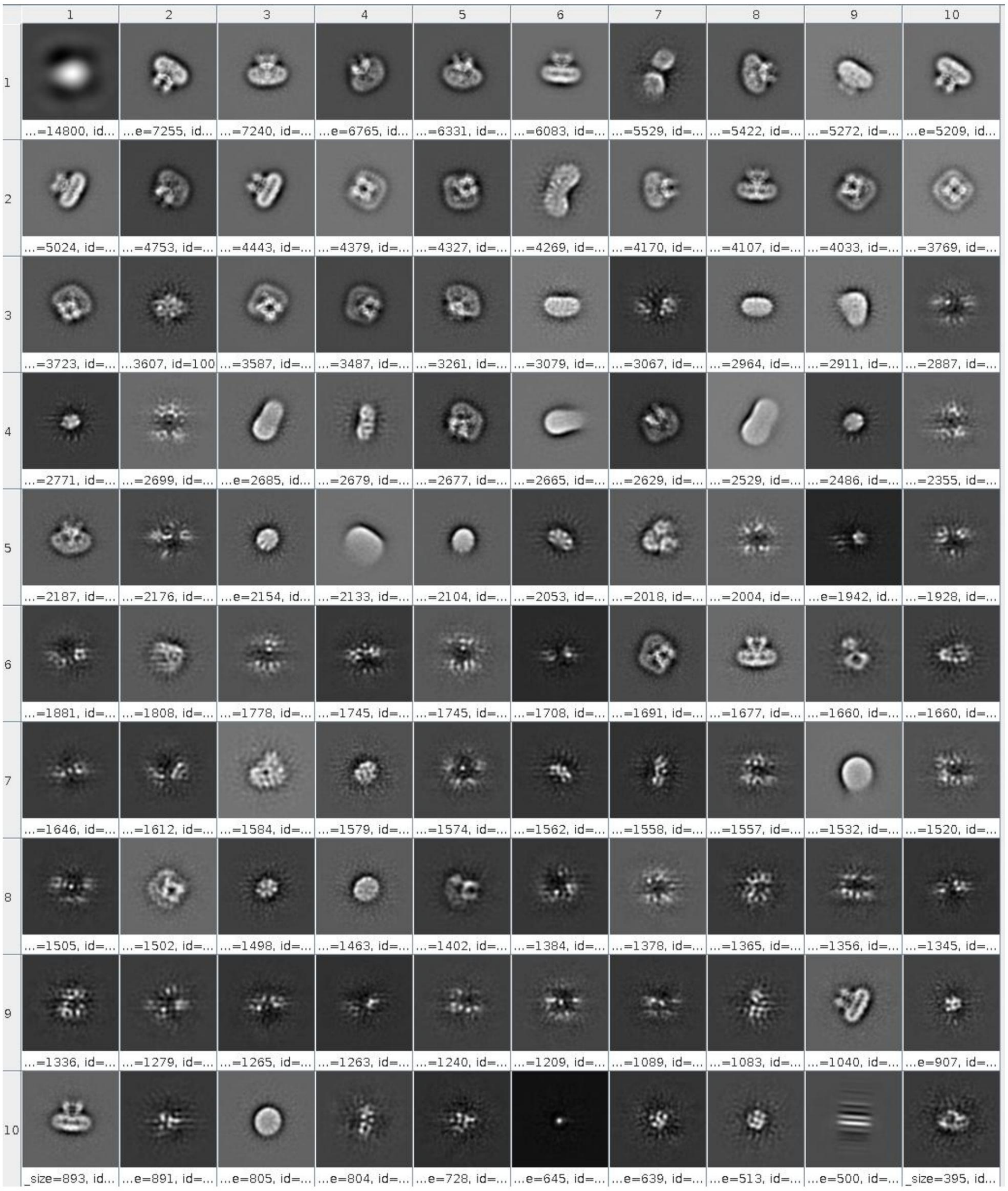

Supplementary Figure S11: Final 2D class averages from the EMPIAR-10081 dataset obtained using AlignPCA-2D.

Supplementary Figure S12

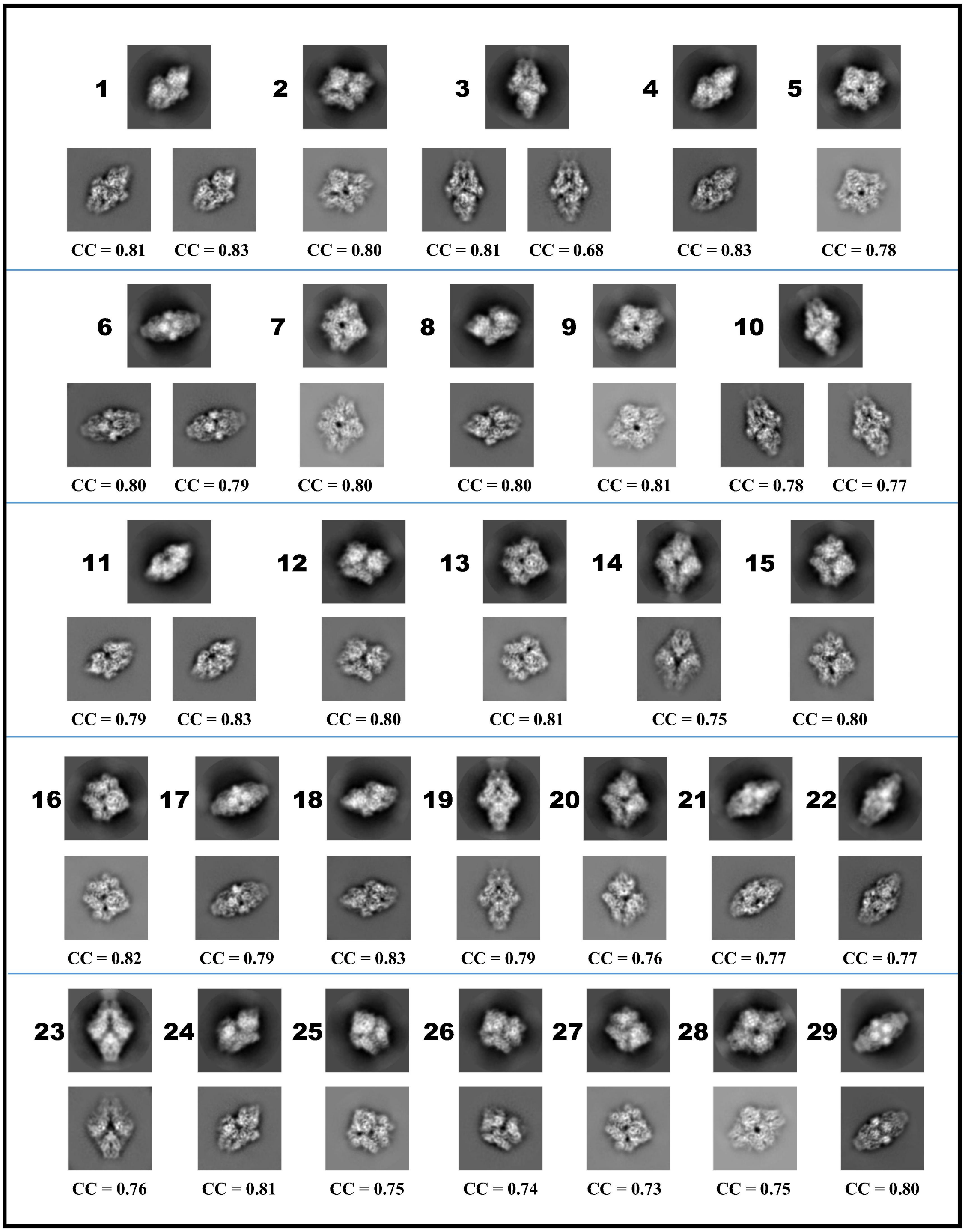

Supplementary Figure S12: Comparison of class averages from AlignPCA-2D and RELION. AlignPCA-2D averages (bottom) were aligned to the corresponding RELION templates (top), and similarity was quantified using cross-correlation (CC). RELION identified 29 structural views, compared to 30 obtained with AlignPCA-2D. Due to structural similarity between some projections, certain classes correspond to multiple classes in the other method.

Supplementary Figure S13

EMPIAR-10061 - CryoSPARC

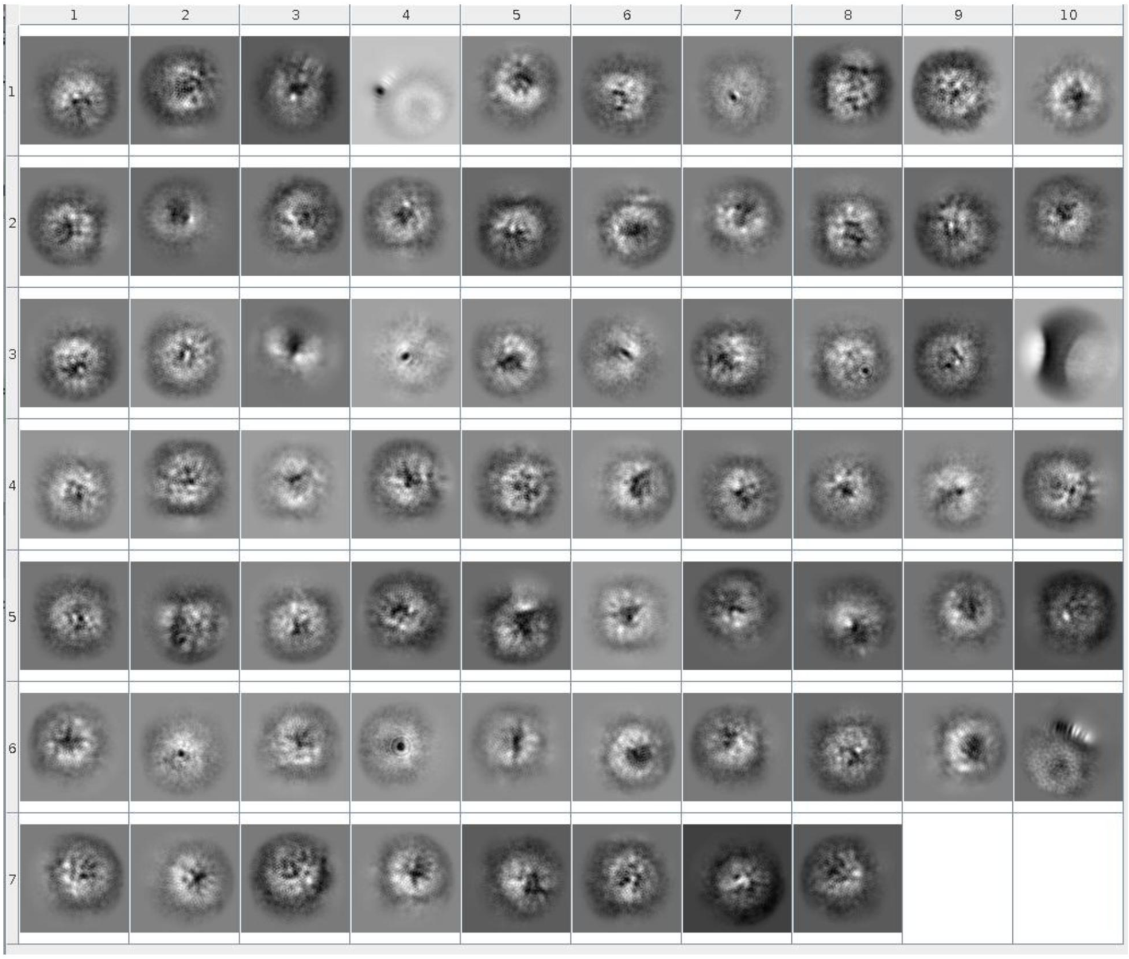

Supplementary Figure S13: Characterization of discarded particles in CryoSPARC. A total of 68 poorly defined classes were identified in the EMPIAR-10061 dataset, representing 212,838 particles.

Supplementary Figure S14

EMPIAR-10061 - rerun CryoSPARC

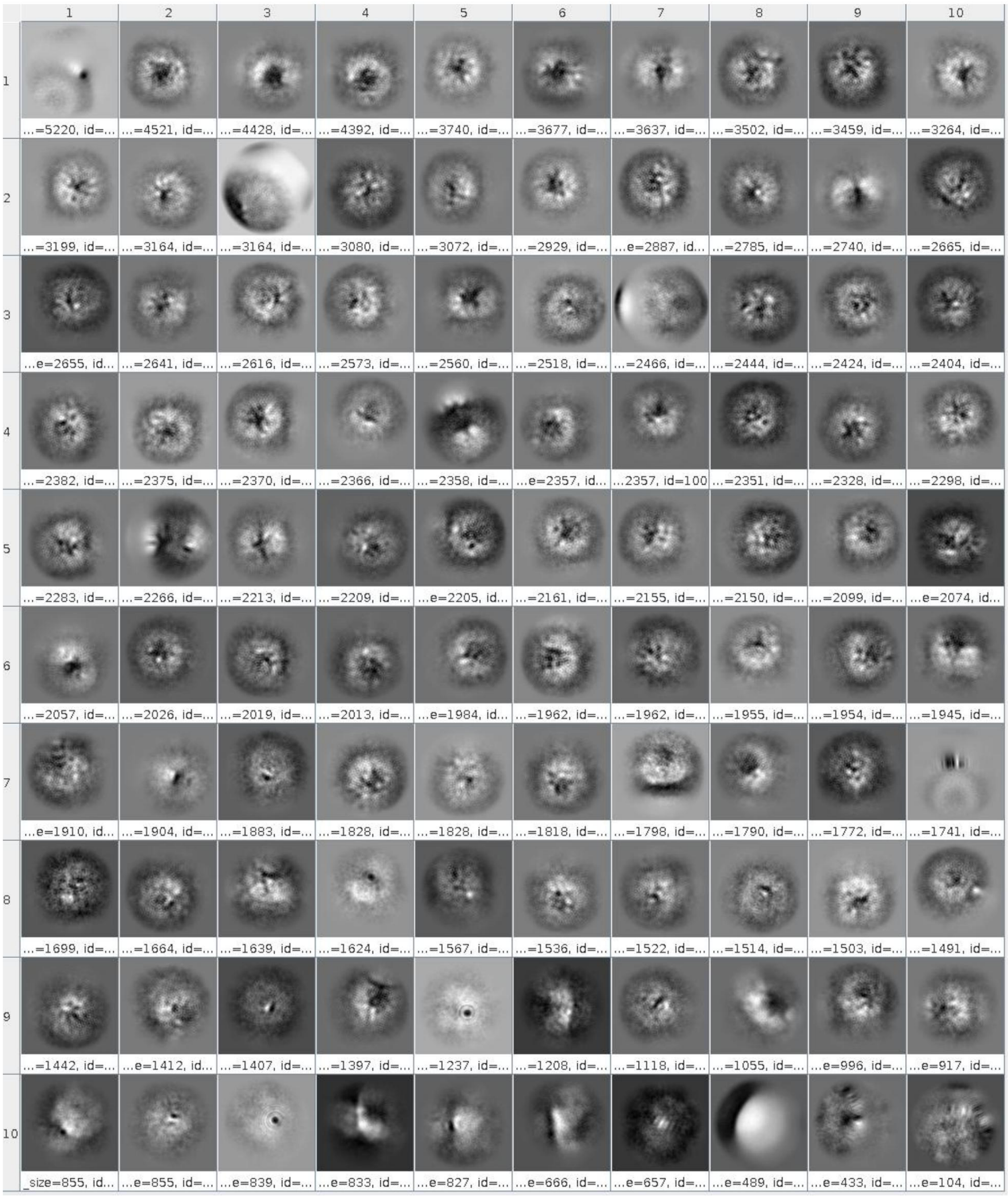

Supplementary Figure S14: Second round of 2D classification in CryoSPARC for dataset EMPIAR-10061. This figure displays the class averages obtained after reprocessing the subset of particles previously identified as poorly defined in Supplementary Figure 13.

Supplementary Figure S15

EMPIAR-10061 - RELION

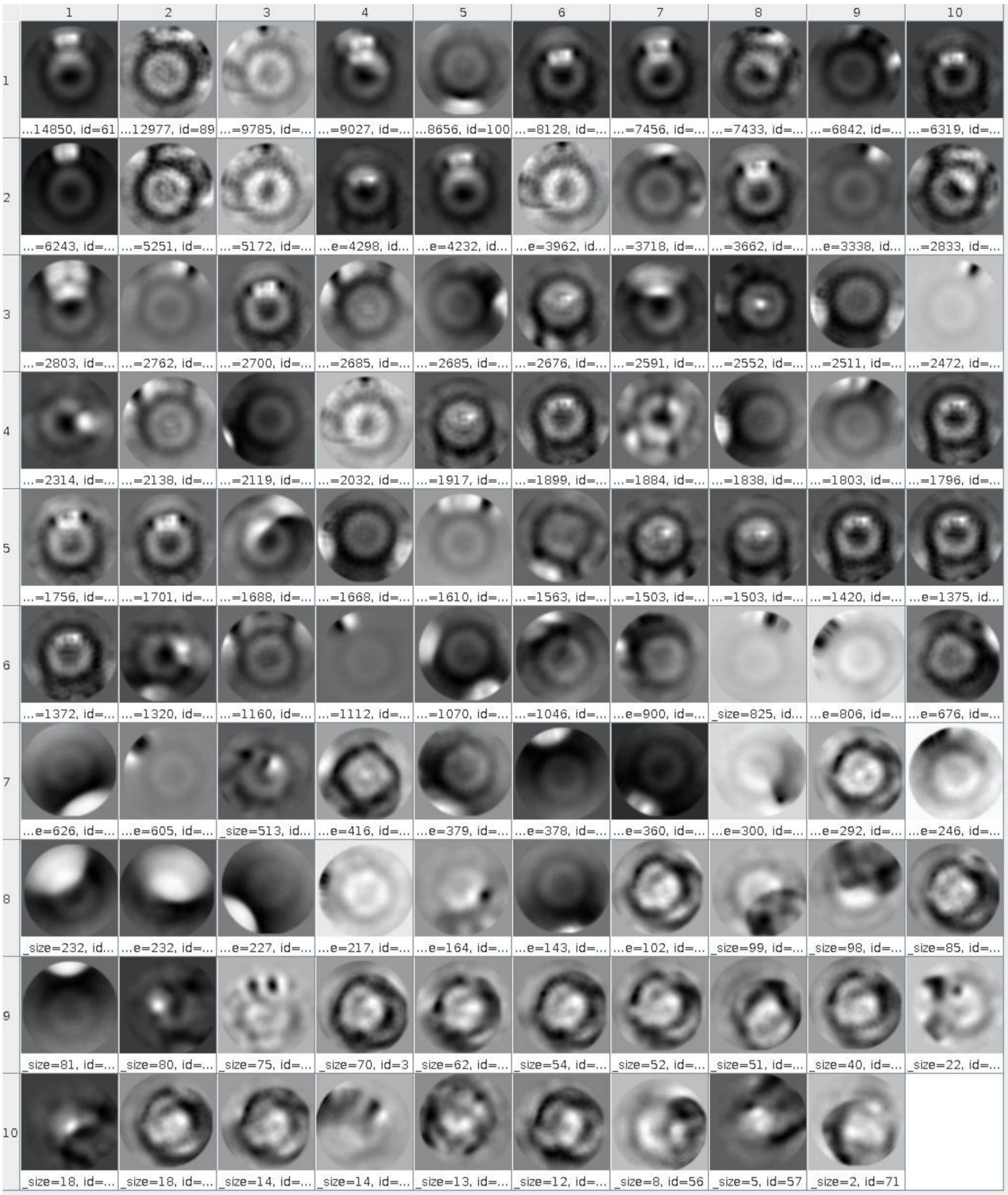

Supplementary Figure S15: Second round of 2D classification with RELION for the EMPIAR-10061 dataset. This figure displays the class averages obtained after reprocessing the subset of particles previously identified as poorly defined by CryoSPARC (as shown in Supplementary Figure 13).

Supplementary Figure S16

EMPIAR-10061 - alignPCA-2D

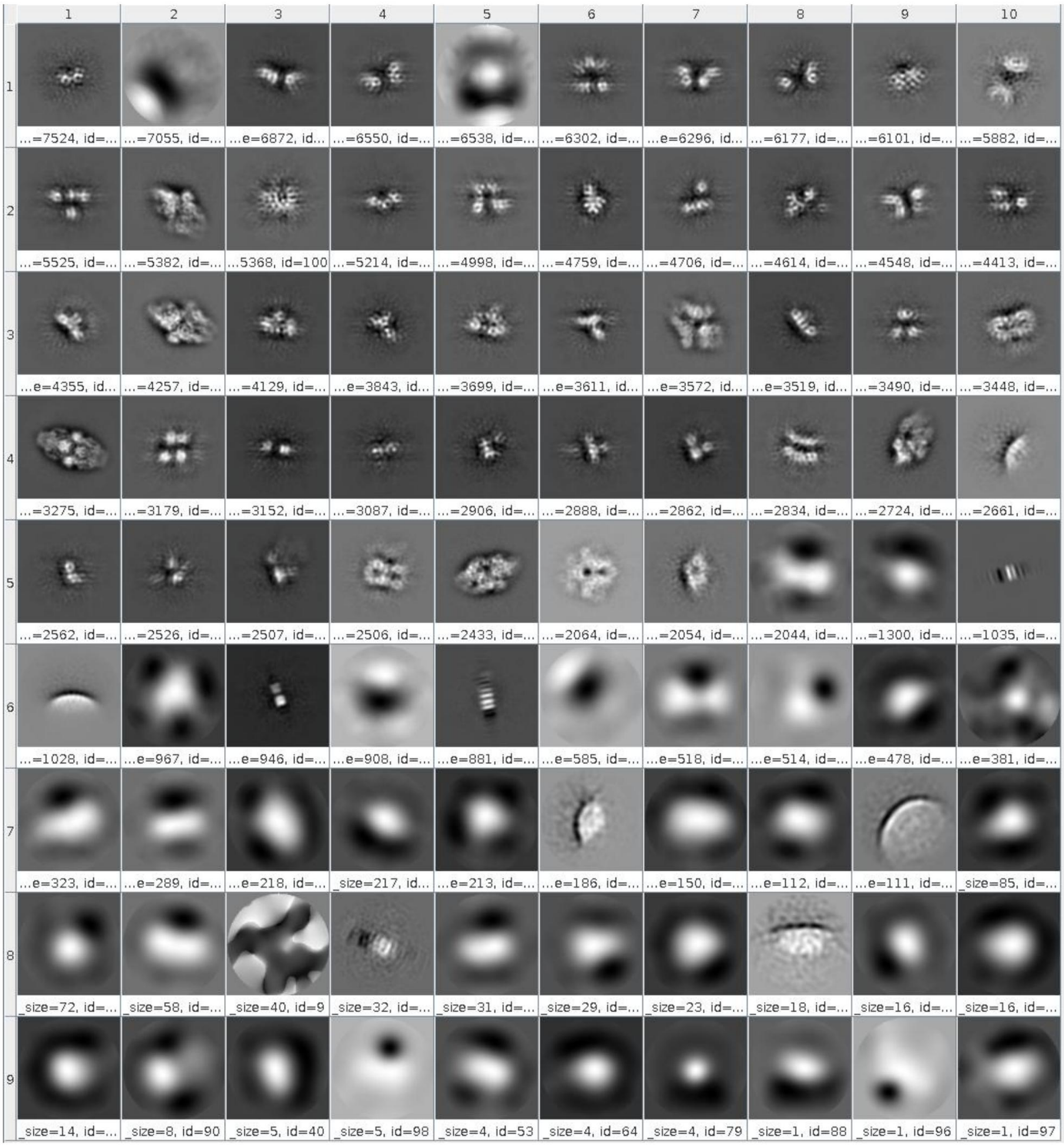

Supplementary Figure S16: Second round of 2D classification with AlignPCA-2D for the EMPIAR-10061 dataset. This figure displays the class averages obtained after reprocessing the subset of particles previously identified as poorly defined by CryoSPARC (as shown in Supplementary Figure 13).

Supplementary Figure S17

EMPIAR-10061 - Align - CryoSPARC

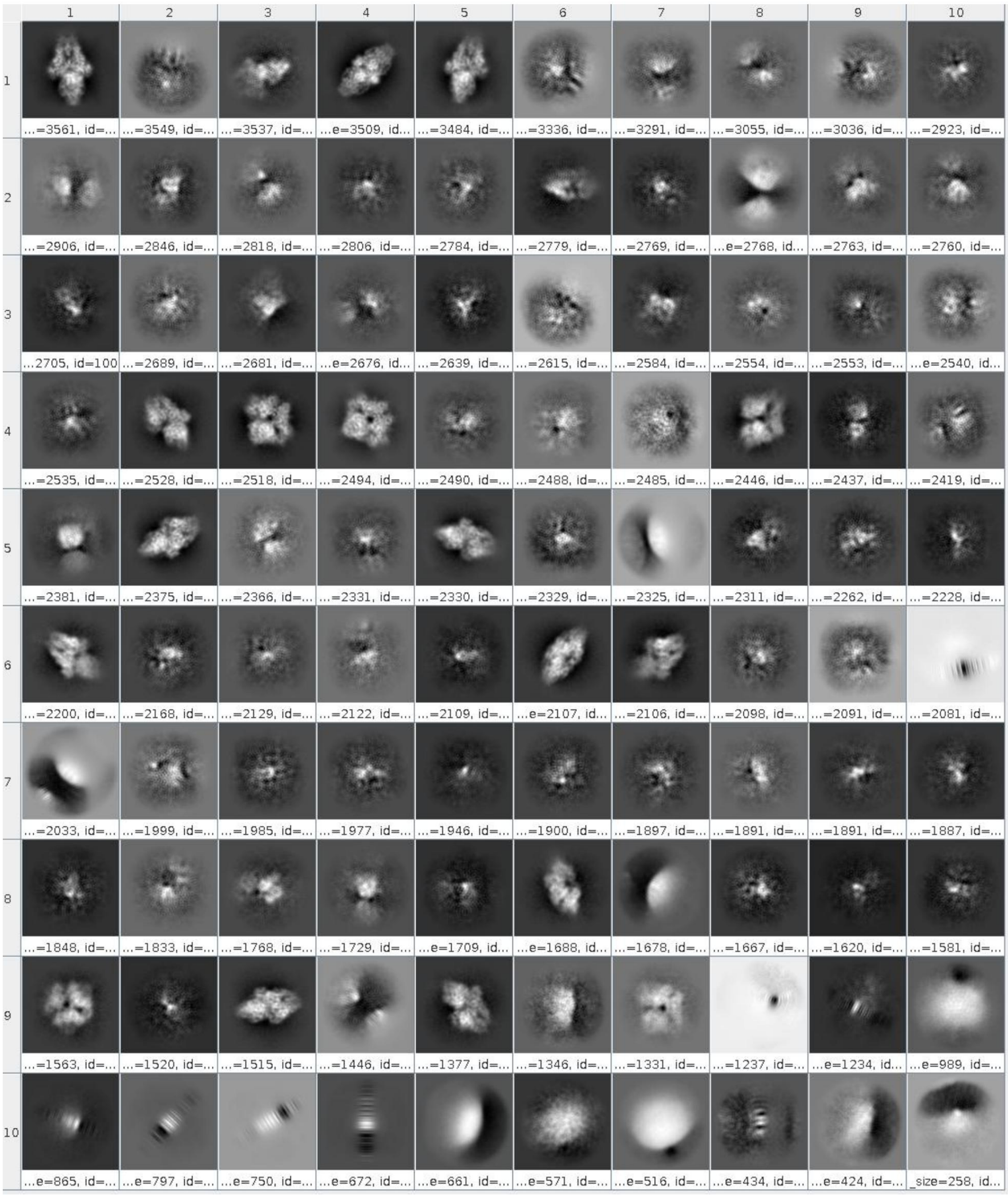

Supplementary Figure S17: Recovery of structural features with CryoSPARC using AlignPCA-2D alignments. The 212,838 particles from the poor-quality classes in Supplementary Figure 13 were re-processed in CryoSPARC. By applying the orientations previously determined by AlignPCA-2D, new 2D class averages were generated, showing improved structural features.

Supplementary Figure S18

EMPIAR-10061 - Align - RELION

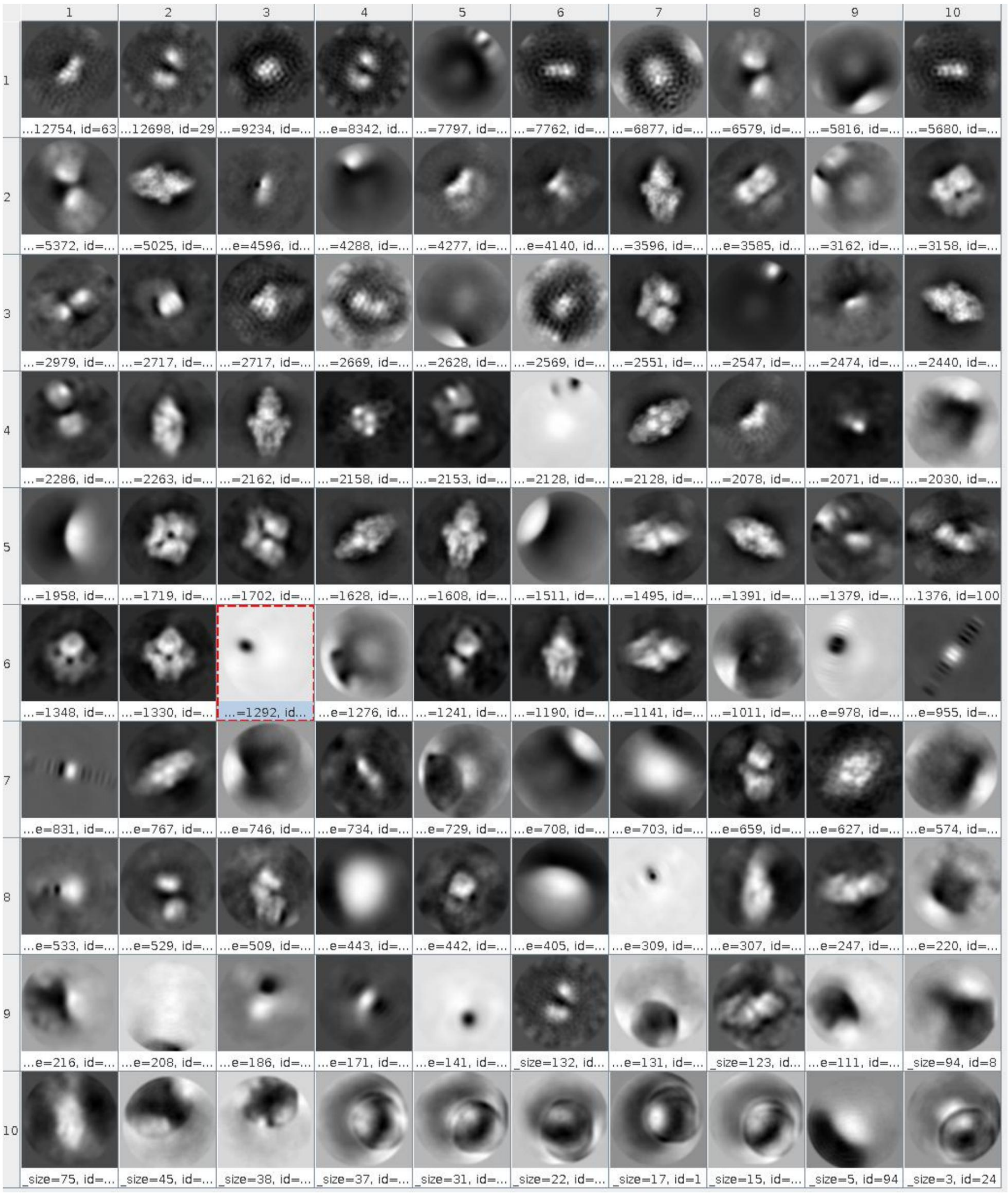

Supplementary Figure S18: Recovery of structural features with RELION using AlignPCA-2D alignments. The 212,838 particles from the poor-quality classes in Supplementary Figure 13 were re-processed in RELION. By applying the orientations previously determined by AlignPCA-2D, new 2D class averages were generated, showing improved structural features.
